# A comprehensive review of *Siraitia grosvenorii* (Swingle) C. Jeffrey: chemical composition, pharmacology, toxicology, status of resources development, and applications

**DOI:** 10.3389/fphar.2024.1388747

**Published:** 2024-04-04

**Authors:** Huaxue Huang, Zhi Peng, Shuang Zhan, Wei Li, Dai Liu, Sirui Huang, Yizhun Zhu, Wei Wang

**Affiliations:** ^1^ School of Pharmacy, Macau University of Science and Technology, Taipa, Macao SAR, China; ^2^ School of Pharmacy, Hunan University of Traditional Chinese Medicine, Changsha, Hunan, China; ^3^ Research and Development Institute of Hunan Huacheng Biotech, Inc., Changsha, Hunan, China; ^4^ Hunan Natural Sweetener Engineering Technology Research Center, Changsha, Hunan, China

**Keywords:** *Siraitia grosvenorii*, mogrosides, chemical compounds, pharmacological effects, toxicological effects, resource development status

## Abstract

*Siraitia grosvenorii* (Swingle) C. Jeffrey (*S. grosvenorii*), a perennial indigenous liana from the Cucurbitaceae family, has historically played a significant role in southern China’s traditional remedies for various ailments. Its dual classification by the Chinese Ministry of Health for both medicinal and food utility underscores its has the potential of versatile applications. Recent research has shed light on the chemical composition, pharmacological effects, and toxicity of *S. grosvenorii*. Its active ingredients include triterpenoids, flavonoids, amino acids, volatile oils, polysaccharides, minerals, vitamins, and other microconstituents. Apart from being a natural sweetener, *S. grosvenorii* has been found to have numerous pharmacological effects, including alleviating cough and phlegm, preventing dental caries, exerting anti-inflammatory and anti-allergic effects, anti-aging and anti-oxidative, hypoglycemic, lipid-lowering, anti-depression, anti-fatigue, anti-schizophrenic, anti-Parkinson, anti-fibrotic, and anti-tumor activities. Despite its versatile potential, there is still a lack of systematic research on *S. grosvenorii* to date. This paper aims to address this gap by providing an overview of the main active components, pharmacological efficacy, toxicity, current status of development and application, development dilemmas, and strategies for intensive exploitation and utilization of *S. grosvenorii*. This paper aims to serve as a guide for researchers and practitioners committed to exploiting the biological resources of *S. grosvenorii* and further exploring its interdisciplinary potential.

## 1 Introduction

**FIGURE 1:**
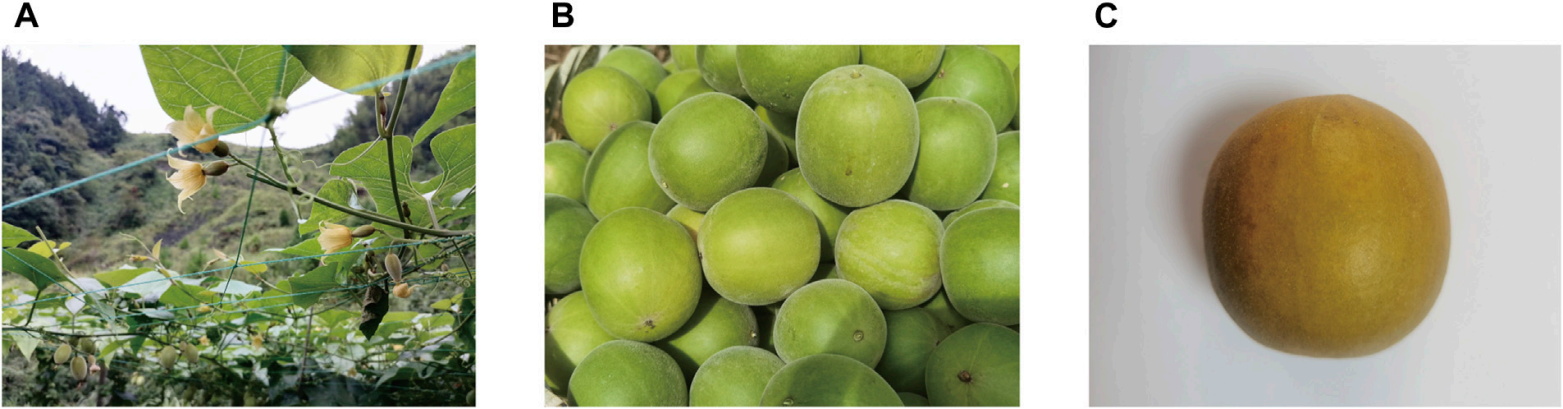
**(A)** The flowers, stems, and leaves of *S. grosvenorii*. **(B)** Fresh fruit of *S. grosvenorii*. **(C)** Dry fruit of *S. grosvenorii*.

*Siraitia grosvenorii* (Swingle) C. Jeffrey (*S. grosvenorii*), stands as an indigenous perennial liana hailing from China within the botanical cohort Cucurbitaceae. Its fruits are commonly known as Luo Han Guo (LHG) or monk fruits (Figure 1). It has an extremely sweet fruit taste and has enjoyed extensive prominence across the southern China since ancient times as a local therapeutic medicine for ailments encompassing the common cold, pharyngitis, and minor gastrointestinal afflictions (Kinghora et al., 1986; Zhang and Li, 2011). Therefore, it was listed by the Chinese Ministry of Health in 1987 in the dual-use category for medicinal and food utility (Li et al., 2014).

The chemical composition of *S. grosvenorii* primarily consists of triterpenoid saponins, flavonoids, amino acids, volatile oils, polysaccharides, minerals, vitamins, and other microconstituents. The main active ingredient in *S. grosvenorii* is mogroside (Mog), which accounts for approximately 3.8% of its content. Mogs are triterpene glucosides, and belong to the cucurbitacinane-type compound category (Jiang et al., 2020; Wei et al., 2023), including Mog III, Mog IV, Mog V, Mog VI, etc., of which Mog V is the main sweet component. Research has shown that extracts obtained from *S. grosvenorii*, as well as its various parts including fruit, root, seed, flower, and leaf, possess beneficial properties. These include the ability to alleviate cough and eliminating phlegm (Liu et al., 2007), mitigate inflammation (Liu et al., 2021b; Liu et al., 2022), regulate blood glucose and lipid levels (Qi et al., 2008), exhibit antioxidant (Zhu et al., 2020) and anti-tumor (Haung et al., 2023) attributes, prevent tooth decay (Zhou et al., 2022a), inhibit fibrosis (Liu et al., 2021a), anti-aging (Nie et al., 2019), among other benefits. Mechanistically, *S. grosvenorii* could regulate physiological activities such as oxidative stress, fatty acid oxidation, and apoptosis, and participate in the alleviation of a variety of diseases, including but not limited to lung, liver, kidney, and brain diseases.

*S. grosvenorii* is a highly valuable economic product that harbors various active ingredients with numerous functions, making it a promising raw material for diverse applications. Currently, the primary application of *S. grosvenorii* is as a sweetener. Although the application of *S. grosvenorii* is promising, there are several issues that need to be addressed, including insufficient product development, low extraction purity, and limited exploration of pharmacological mechanisms. This overview provides a systematic examination of the bioactive constituents, pharmacological effects, toxicological effects, resource development status, practical applications, and strategies for intensive exploitation and utilization of *S. grosvenorii*. It aims to deepen public understanding of this valuable traditional Chinese medicine and provide new perspectives for further investigation into its functional activities and in-depth development of its applications. This comprehensive review seeks to offer new ideas and reference materials to advance the development and utilization of *S. grosvenorii*.

## 2 Chemical composition

No less than 131 triterpenoids, 31 flavonoids, 27 amino acids, 19 volatiles, 6 polysaccharides, 19 minerals, and 4 vitamins have been identified in *S. grosvenorii* (Pandey and Chauhan, 2019, p.; Wu et al., 2022a). Among them, cucurbitanes are the most dominant structural types in *S. grosvenorii*, belonging to the tetracyclic triterpenoids.

### 2.1 Triterpenoids

**FIGURE 2:**
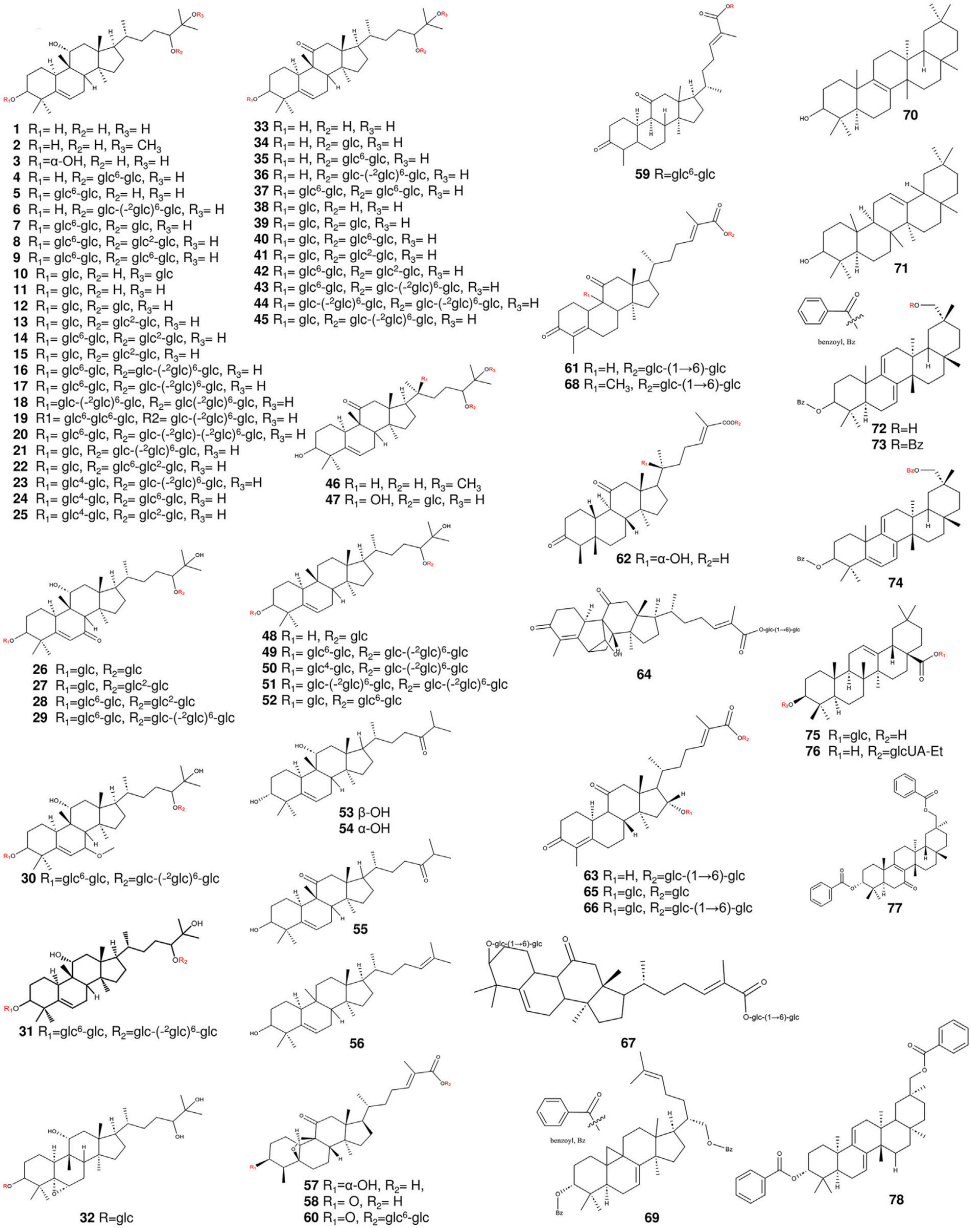
The structures of triterpenoids (compounds 1–78) from *S. grosvenorii*.

**FIGURE 3:**
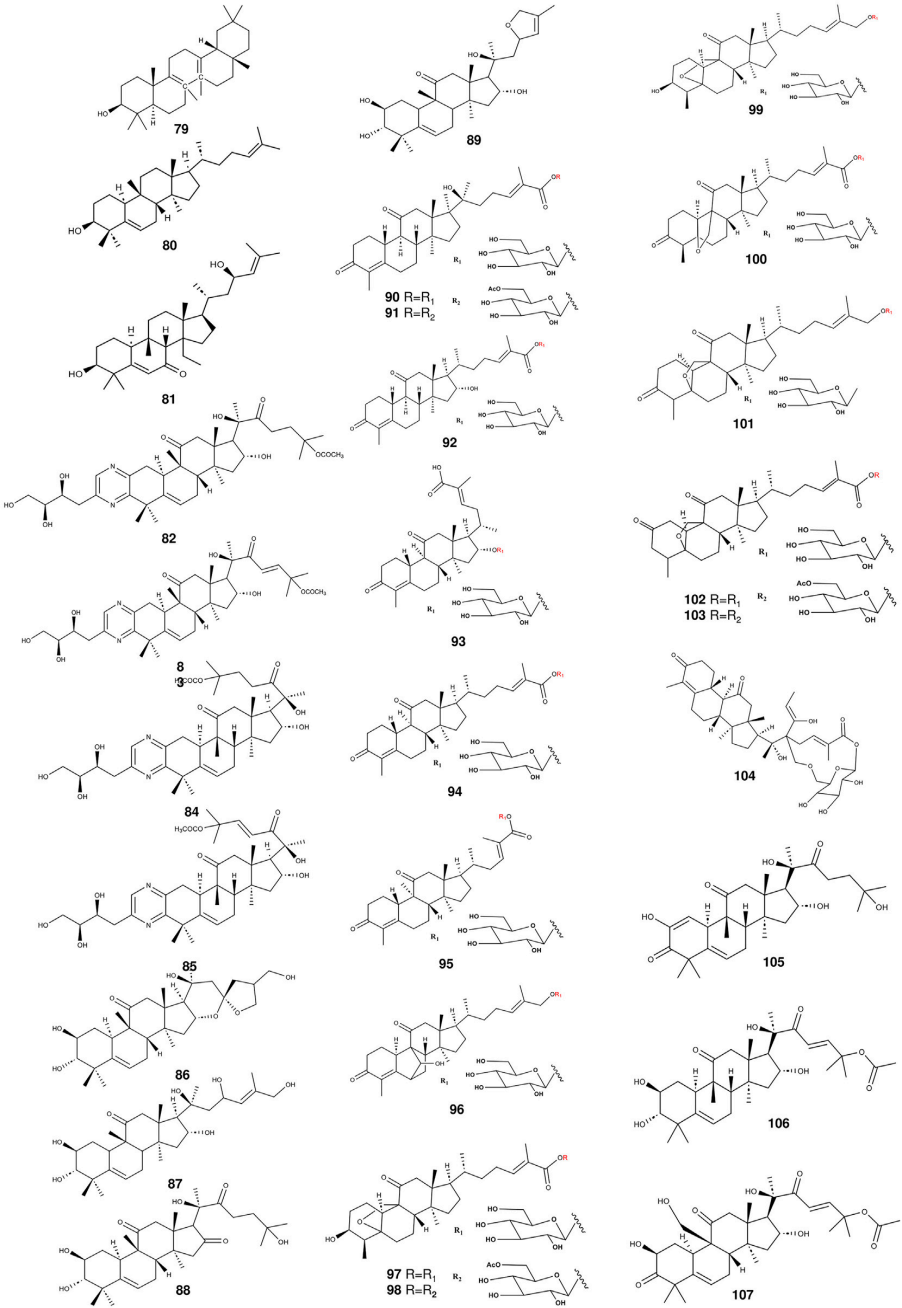
The structures of triterpenoids (compounds 79–107) from *S. grosvenorii*.

**FIGURE 4:**
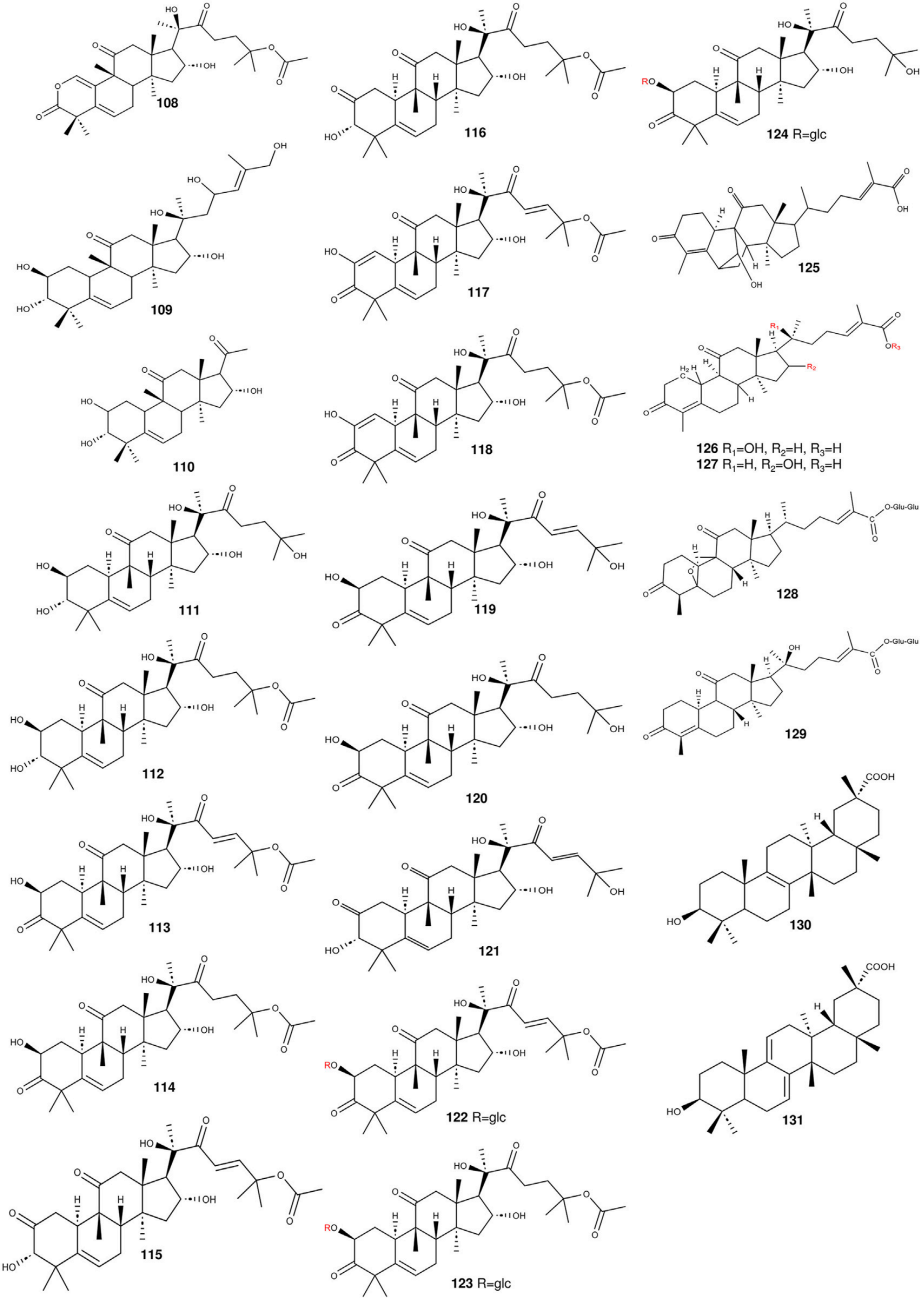
The structures of triterpenoids (compounds 108–131) from *S. grosvenorii*.

The triterpenoid compounds found in *S. grosvenorii* are primarily derivatives of cucurbitane-type tetracyclic triterpenoids, and a variety of Mogs have been isolated and characterized. The total content of Mogs in the fruit of *S. grosvenorii* are approximately 3.8% (Jiang et al., 2020; Wei et al., 2023) (Table 1) (Figures 2–4). Among these Mog compounds, Mog V had the highest content, accounting for about 0.8%–1.3% (w/w) of the fruit’s composition (Li et al., 2014). It possesses an incredibly sweet taste, with a sweetness potency surpassing that of sucrose by a factor of 425 when present at a concentration of 1/10000 (Li et al., 2014). All Mog compounds contain mogrol, [10-cucurbit-5-ene-3,11,24R,25-tetraol], attached to different numbers of glucose units. Categorization of mogrol, according to the conformational changes in the presence or absence of carbonyl group at C-7 position, hydroxyl group at C-11 position, and methyl group at C-25 position, categorized into five groups (Wu et al., 2022a).

**TABLE 1:** The main triterpenoids (3.8%) from *S. grosvenorii*.

No.	Compound name	Origins	References
1	mogrol	fruit	Takemoto et al. (1983)
2	25-methoxymogrol	fruit	Chen et al. (2011)
3	3α-hydroxymogrol	fruit	Chen et al. (2011)
4	mogroside IIA1	fruit	Akihisa et al. (2007)
5	mogroside IIA2	fruit	(Suzuki et al., 2005; Li et al., 2017)
6	mogroside IIIA1	fruit	Li et al. (2017)
7	mogroside IIIA2	fruit	Takemoto et al. (1983), Akihisa et al. (2007)
8	mogroside IV	fruit	Takemoto et al. (1983)
9	mogroside IVA	fruit	Takemoto et al. (1983), Li et al. (2006a)
10	mogroside IIB	fruit	Akihisa et al. (2007)
11	mogroside IE1	fruit	Takemoto et al. (1983)
12	mogroside IIE	fruit	(Wang et al., 2014; Cai et al., 2021)
13	mogroside IIIE	fruit	Takemoto et al. (1983)
14	mogroside IVE	fruit	(Jia and Yang, 2009; Cao et al., 2018)
15	mogroside III	fruit	Qin et al. (2014)
16	mogroside V	fruit	Takemoto et al. (1983)
17	mogroside VA1	fruit	Chaturvedula and Meneni (2015)
18	mogroside VI	fruit	Takemoto et al. (1983)
19	mogroside VI A	fruit	Niu et al. (2017)
20	mogroside VI B	fruit	Niu et al. (2017)
21	siamenoside I	fruit	Jia and Yang (2009)
22	grosmomoside I	fruit	Yang et al. (2005)
23	isomogroside V	fruit	Jia and Yang (2009)
24	isomogroside IVa	fruit	Li et al. (2017)
25	isomogroside IVe	fruit	Li et al. (2017)
26	7-oxo-mogroside IIE	fruit	He et al. (2017)
27	7-oxo-mogroside IIIE	fruit	Niu et al. (2017)
28	7-oxo-mogroside IV	fruit	Niu et al. (2017)
29	7-oxo-mogroside V	fruit	He et al. (2017)
30	7β-methoxy-mogroside V	fruit	Chu et al. (2019)
31	11-epimogroside V	fruit	Jia and Yang (2009)
32	5α,6α-epoxymogroside IE1	fruit	Jia and Yang (2009)
33	11-oxo-mogrol	fruit	Ju et al. (2020)
34	11-oxo-mogroside IA1	fruit	Li et al. (2006a)
35	11-oxomogroside IIA1	fruit	Jia and Yang (2009)
36	11-oxo-mogroside IIIA1	fruit	Chu et al. (2019)
37	11-oxo-mogroside IVA	fruit	Jia and Yang (2009)
38	11-oxo-mogroside IE1	fruit	Takemoto et al. (1983)
39	11-oxo-mogroside IIE	fruit	Li et al. (2006a)
40	11-oxo-mogroside III	fruit	Li et al. (2007)
41	11-oxo-mogroside IIIE	fruit	Niu et al. (2017)
42	11-oxo-mogroside IV	fruit	Niu et al. (2017)
43	11-oxo-mogroside V	fruit	Jia and Yang (2009)
44	11-oxo-mogroside VI	fruit	Li et al. (2017)
45	11-oxo-siamenoside I	fruit	Li et al. (2017)
46	25-methoxy-11-oxomogrol	fruit	Chen et al. (2011)
47	20-hydroxy-11-oxomogroside I A1	fruit	Li et al. (2006a)
48	mogroside IA	fruit	Takemoto et al. (1983)
49	11-deoxymogroside V	fruit	Li et al. (2017)
50	11-deoxyisomogroside V	fruit	Prakash and Chaturvedula (2014)
51	11-deoxymogroside VI	fruit	Prakash and Chaturvedula (2014), Fang et al. (2017)
52	11-deoxymogroside III	fruit	Akihisa et al. (2007), Fang et al. (2017)
53	25-dehydroxy-24-oxomogrol	fruit	Chen et al. (2011)
54	3-hydroxy-25-dehydroxy-24oxomogrol	fruit	Chen et al. (2011)
55	bryogenin	fruit	Chen et al. (2011)
56	10α-cucurbitadienol	seed oil	Jia and Yang (2009)
57	siraitic acid A	root	Wang et al. (1996)
58	siraitic acid B	root	Wang et al. (1996)
59	siraitic acid IIA	root	Li et al. (2009), Lu et al. (2023a)
60	siraitic acid IIB	root	Li et al. (2009)
61	siraitic acid IIC	root	Li et al. (2009)
62	siraitic acid D	root	Wang (2023)
63	siraitic acid IIE	root	Lu et al. (2023a)
64	siraitic acid IIF	root	Lu et al. (2023a)
65	siraitic acid IIBE	root	Lu et al. (2023a)
66	siraitic acid IIIE	root	Lu et al. (2023a)
67	siraitic acid IVH	root	Lu et al. (2023a)
68	siraitic acid IIG	root	Lu et al. (2023a)
69	mogroseter	leaf	Li et al. (2003)
70	oleanolanes	seed, root	Li et al. (2003)
71	β-amyrin	fruit	Wang et al. (2015)
72	karounidiol 3-benzoate	fruit	Ukiya et al. (2002)
73	karounidiol dibenzoate	flower	Ukiya et al. (2002)
74	5-dehydrokarounidiol dibenzoate	fruit	Lim (2012)
75	oleanolic acid 28 -O-β-D-glucopyranoside	fruit	Wu et al. (2023)
76	oleanolic acid 3-O-β-D-pyranglucuronide −6 ′-ethyl ester	fruit	Wu et al. (2023)
77	(3α)-3,29-dihydroxy-7-oxomultiflor-8-ene-3,29-diyl dibenzoate	fruit	Wu et al. (2023)
78	3, 29-O-dibenzoyloxykarounidiol	fruit	Wu et al. (2023)
79	isomultiflorenol	fruit	Wu et al. (2023)
80	cucurbitane-5, 24-diene-3-β-ol	fruit	Wu et al. (2023)
81	balsaminol E	fruit	Wu et al. (2023)
82	siragrosvenin A	root	Wang et al., 2022; Wang (2023)
83	siragrosvenin B	root	Wang et al., 2022; Wang (2023)
84	siragrosvenin C	root	Wang et al., 2022; Wang (2023)
85	siragrosvenin D	root	Wang et al., 2022; Wang (2023)
86	siragrosvenin E	root	Wang (2023)
87	siragrosvenin F	root	Wang (2023)
88	siragrosvenin G	root	Wang (2023)
89	siragrosvenin H	root	Wang (2023)
90	siraitiaoside A	root	Wang (2023)
91	siraitiaoside B	root	Wang (2023)
92	siraitiaoside C	root	Wang (2023)
93	siraitiaoside D	root	Wang (2023)
94	siraitiaoside E	root	Wang (2023)
95	siraitiaoside F	root	Wang (2023)
96	siraitiaoside G	root	Wang (2023)
97	siraitiaoside H	root	Wang (2023)
98	siraitiaoside I	root	Wang (2023)
99	siraitiaoside J	root	Wang (2023)
100	siraitiaoside K	root	Wang (2023)
101	siraitiaoside L	root	Wang (2023)
102	siraitiaoside M	root	Wang (2023)
103	siraitiaoside N	root	Wang (2023)
104	siraitiaoside O	root	Wang (2023)
105	cucurbitacin L	root	Wang (2023)
106	cucurbitacin Q1	root	Wang (2023)
107	cucurbitacin A	root	Wang (2023)
108	neocucurbitacin D	root	Wang (2023)
109	jinfushanencin F	root	Wang (2023)
110	2,3,16-trihydroxy-4,4,9,14tetramethyl-19-norpregn-5-ene1,20-dione	root	Wang (2023)
111	23,24-dihydrocucurbitacin F	root	Wang (2023)
112	cucurbitacin IIA	root	Wang (2023)
113	cucurbitacin B	root	Wang (2023)
114	23,24-dihydrocucurbitacin B	root	Wang (2023)
115	isocucurbitacin B	root	Wang (2023)
116	23,24-dihydroisocucurbitacin B	root	Wang (2023)
117	cucurbitacin E	root	Wang (2023)
118	23,24-dihydrocucurbitacin E	root	Wang (2023)
119	cucurbitacin D	root	Wang (2023)
120	23,24-dihydrocucurbitacin D	root	Wang (2023)
121	isocucurbitacin D	root	Wang (2023)
122	arvenin I	root	Wang (2023)
123	arvenin II	root	Wang (2023)
124	arvenin IV	root	Wang (2023)
125	siraitic acid C	root	(Si et al., 2005; Wang, 2023)
126	siraitic acid E	root	Wang (2023)
127	siraitic acid F	root	(Si et al., 2005; Wang, 2023)
128	5β,19β-epoxy-29-nor-3,11-dioxo-cucurbit-24-ene-27-oic acid 27-O-β-D-glucopyranosyl-(1→6)-β-D-glucopyranoside	root	Wang (2023)
129	19,29-nor-3,11-dix-cucurbit-4,24-diene-27-oic acid 27-O-β-D-glucopyranosyl-(1→6)-β-D-glucopyranoside	root	Wang (2023)
130	bryonolic acid	root	Wang (2023)
131	karounidin acid	root	Wang (2023)

There are studies show that the sweetness of 3β-hydroxy-cucurbit-5-ene derivative glycosides depends on the count and arrangement of glucose units, the oxygen function group at position C-11, and the side chain hydroxylation (Kasai et al., 1988). Glycosides possessing at least three glucose units are endowed with sweetness. For instance, Mog IIE, Mog IIIE, and Mog III with fewer than three glucose units, lack sweet taste (Kasai et al., 1988). Whereas as Siamenoside I, containing 5 glucose units, standing as the most intensely sweet triterpenoid glycoside isolated to date (Li et al., 2014). However, the relationship between glucose unit number and sweetness is not necessarily proportional. For example, despite having the same number of glucose units as Siamenoside I, Mog IVA and Mog IVE are not as sweet (Wu et al., 2022a). Intriguingly, recent study discloses that the glycosyltransferase UGT94-289-3 can catalyze the glycosylation of bitter Mog IIE and III during fruit post-ripening to produce sweet Mog containing 4 to 6 glucose units (Cui et al., 2023). Under optimized catalytic conditions, the glycosyltransferase UGT94-289-3 can effectuate the conversion of 95% of Mog into sweet Mog using Mog III as substrate (Cui et al., 2023). Another pivotal determinant of sweetness is the oxygen functional group at the C-11 position of the aglycone moiet. Mog IVA and Mog IVE containing 11α-hydroxyl glycosides tasted sweet, in stark contrast to compounds with 11β-hydroxyl glycosides that were tasteless, and compounds containing 11-oxides and their dehydrogenated derivatives taste bitter (Pawar et al., 2013). Sweetness is further influenced by side chain hydroxylation. For example, the bitter 11-oxo-mogrosides (11-O-Mog) can be sweetened by hydroxylation of the side-chain double bond with osmium tetroxide (Wu et al., 2022a).

It merits attention that the content of Mogs within the fruit undergoes variations corresponding to its developmental stages. During the initial 30 days post-pollination, Mog IIE predominates, gradually yielding to elevated concentrations of Mog III between 30 and 55 days. Subsequent phases from 56 to 70 days witness the emergence of Mog IV, Mog IVA, and Mog IVE, accompanied a change in fruit flavor from bitter to sweet. Mog V was produced at 70 days, peaking in its sweetness around 85 days post-pollination (Shivani et al., 2021).

### 2.2 Flavonoids

**FIGURE 5:**
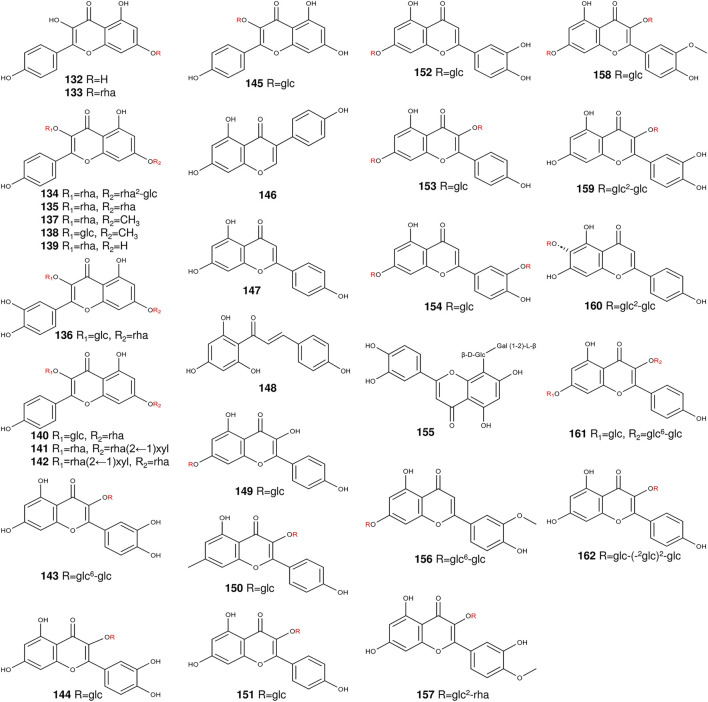
The structures of flavonoids from *S. grosvenorii*.

Flavonoids are a branch of a class of polyphenols with the structure of benzo-γ-pyrrodone (Dixon et al., 1983). Among the compounds scrutinized, grosvenorine emerged as the principal flavonoid glycoside of the fruits of *S. grosvenorii* (Wu et al., 2022a) (Table 2) (Figure 5). Notably, among grosvenorine metabolites, were kaempferol and quercetin, emerging as the predominant flavonoids within *S. grosvenorii* (Duan et al., 2023). In *S. grosvenorii*, flavonol glycoside content varied with time. Commencing at day 40 and culminating at day 50, a rapid augmentation in flavonol glycosides was manifest. It peaks around day 50, declines after day 60, and eventually stabilizes to around day 20 (Li et al., 2014).

**TABLE 2:** The main flavonoids from *S. grosvenorii*.

No.	Compound name	Origins	References
132	kaempferol	flower, leaf, fruit	(Yang et al., 2008; Fang et al., 2017)
133	kaempferol-7-O-α-L-rhamnopyranoside	flower, leaf, friut	Wu et al. (2023)
134	grosvenorine	flower, leaf, friut	Wang et al. (2015), Sung et al. (2020)
135	kaempferitrin	leaf, fruit	Wu et al. (2023)
136	quercetin 3-O-β-D-glucopyranosyl 7-O-α-L-rhamnopyranoside	leaf, fruit	(Si et al., 1994; Chen et al., 2006)
137	7-methoxy-kaempferol 3-O-α-L-rhamnopyranoside	flower	(Si et al., 1994; Chen et al., 2006)
138	7-methoxykaempferol 3-O-β-D-glucopyranoside	flower	Mo and Li (2009)
139	afzelin	fruit	Mo and Li (2009)
140	kaempferol-3-O-β-D-glucopyranosyl-7-O-α-L-rhamnopyranoside	fruit	Wu et al. (2023)
141	kaempferia galanga phenol-3-O-α-L-buckthorn indican-7-O-β-D-wood glycoside (1→2)-O-α-L - buckthorn glycoside	fruit	Wu et al. (2023)
142	sagittatin A	fruit	Wu et al. (2023)
143	rutin	fruit	Fang et al. (2017)
144	quercitrin	fruit	Fang et al. (2017)
145	kaempferol 3-O-β-rutinoside	fruit	Fang et al. (2017)
146	genistein	fruit	Fang et al. (2017)
147	apigenin	fruit	Zhou et al. (2022a)
148	naringenin chalcone	fruit	Zhou et al. (2022a)
149	kaempferol-7-O-rhamnoside	fruit	Zhou et al. (2022a)
150	kaempferol-3-O-rhamnoside (Afzelin) (Kaempferin)	fruit	Zhou et al. (2022a)
151	kaempferol-3-O-glucoside (Astragalin)	fruit	Zhou et al. (2022a)
152	luteolin-7-O-glucoside (Cynaroside)	fruit	Zhou et al. (2022a)
153	kaempferol-3,7-O-dirhamnoside (Kaempferitrin)	fruit	Zhou et al. (2022a)
154	luteolin-7,3′-di-O-glucoside	fruit	Zhou et al. (2022a)
155	orientin-2″-O-galactoside	fruit	Zhou et al. (2022a)
156	chrysoeriol-7-O-gentiobioside	fruit	Zhou et al. (2022a)
157	isorhamnetin-3-O-neohesperidoside	fruit	Zhou et al. (2022a)
158	isorhamnetin-3-O-glucoside-7-O-rhamnoside	fruit	Zhou et al. (2022a)
159	quercetin-3-O-sophoroside (Baimaside)	fruit	Zhou et al. (2022a)
160	isovitexin-2″-O-(6‴-p-coumaroyl) glucoside	fruit	Zhou et al. (2022a)
161	kaempferol-3-O-robinoside-7-O-rhamnoside (Robinin)	fruit	Zhou et al. (2022a)
162	kaempferol-3-O-sophorotrioside	fruit	Zhou et al. (2022a)

### 2.3 Amino acids

**FIGURE 6:**
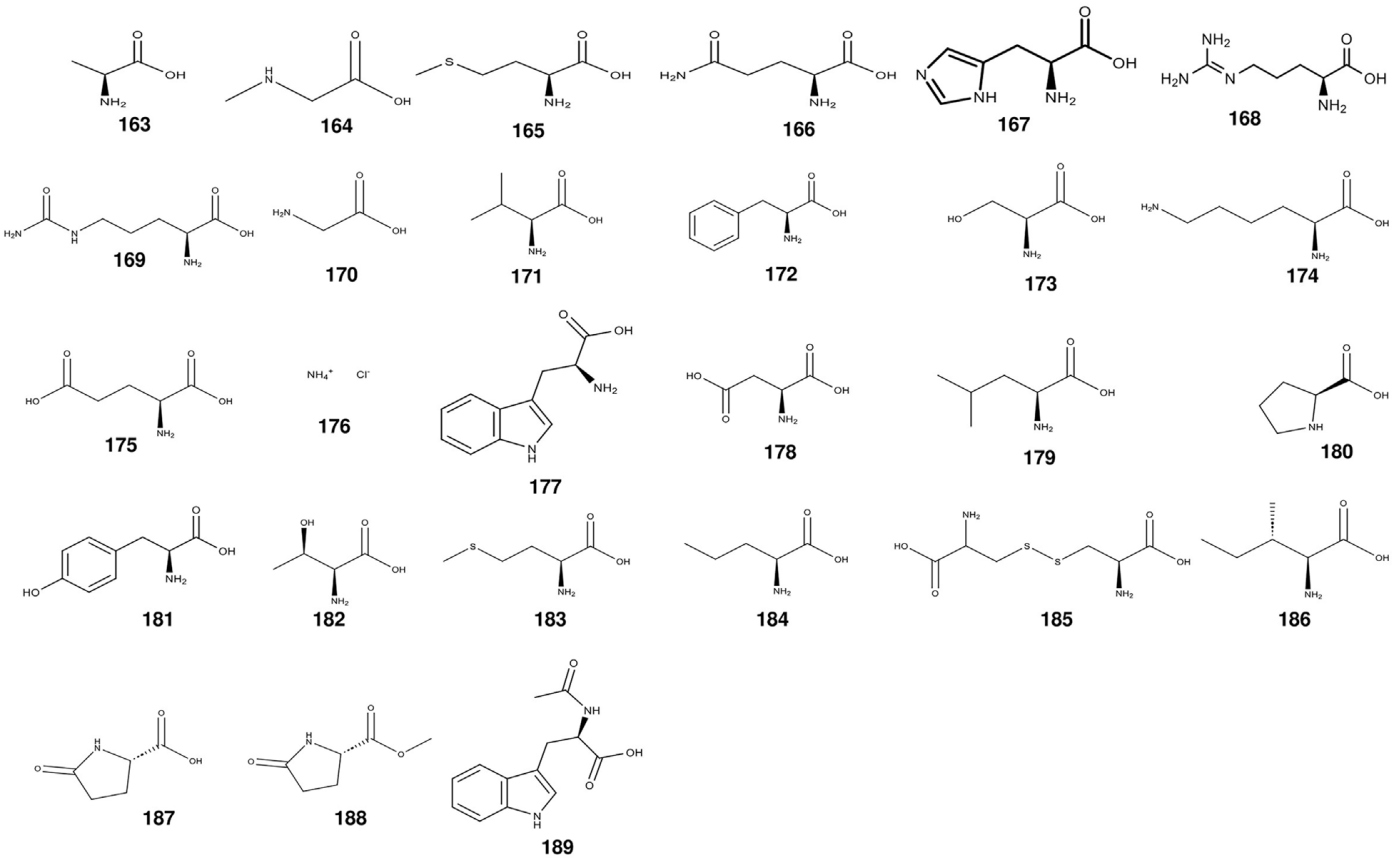
The structures of amino acids from *S. grosvenorii*.

Free amino acids, pivotal contributors to protein synthesis, cell signaling, metabolism, physiology, and health. In the pursuit of comprehending amino acid distributions within *S. grosvenorii*, investigations have delved into the protein content of both fresh and dried fruits (Table 3) (Figure 6), revealing ranges of 8.67%–13.35% and 7.1%–7.8%, respectively. However, the efficiency of amino acid extraction, especially the yield of the ultrasonic methodologies coupled with chromatographic separation, was only 6.54% (Liu et al., 2011). Of the 23 amino acids identified in *S. grosvenorii*, 19 were significantly increased after Nano-selenium treatment. Including histidine (108.7%), tryptophan (104.5%), serine (87.4%), glycine (85.9%), lysine (67.5%), tyrosine (78.4%), aspartic acid (65.2%), phenylalanine (60.1%), valine (62.0%), glutamic acid (53.4%) and proline (52.5%) (Zhou et al., 2022a). Furthermore, discerning the preponderant amino acids in *S. grosvenorii*, glutamic acid and aspartic acid emerged as the most abundant constituents, underscoring their pivotal roles in the botanical matrix (Duan et al., 2023).

**TABLE 3:** The main amino acids from *S. grosvenorii*.

No.	Compound name	Origins	References
163	alanine	fruit	Xu et al. (2022b)
164	sarcosine	fruit	Liu et al. (2022)
165	aminobutyric acid	fruit	Zhou et al. (2022a)
166	glutamine	fruit	Fang et al. (2017)
167	histidine	fruit	Fang et al. (2017)
168	arginine	fruit	Fang et al. (2017)
169	citrulline	fruit	Fang et al. (2017)
170	glycine	fruit	Xu et al. (2022b)
171	valine	fruit	Fang et al. (2017)
172	phenylalanine	fruit	Fang et al. (2017)
173	serine	fruit	Luo et al. (2016)
174	lysine	fruit	Wang et al. (2023)
175	glutamic acid	fruit	Fang et al. (2017)
176	ammonium chloride	fruit	Zhou et al. (2022a)
177	tryptophan	fruit	Fang et al. (2017)
178	aspartic acid	fruit	Xu and Meng (1986)
179	leucine	fruit	Fang et al. (2017)
180	proline	fruit	Fang et al. (2017)
181	tyrosine	fruit	Fang et al. (2017)
182	threonine	fruit	Zhou et al. (2022a)
183	methionine	fruit	Fang et al. (2017)
184	norvaline	fruit	Fang et al. (2017)
185	cystine	fruit	Zhang et al. (2020b)
186	isoleucine	fruit	Zhang et al. (2020b)
187	L pyroglutamic acid	root	Wang (2023)
188	L Methyl pyroglutamic acid	root	Wang (2023)
189	N-acetyl-D-tryptophan	fruit	Fang et al. (2017)

### 2.4 Volatile oils

**FIGURE 7:**
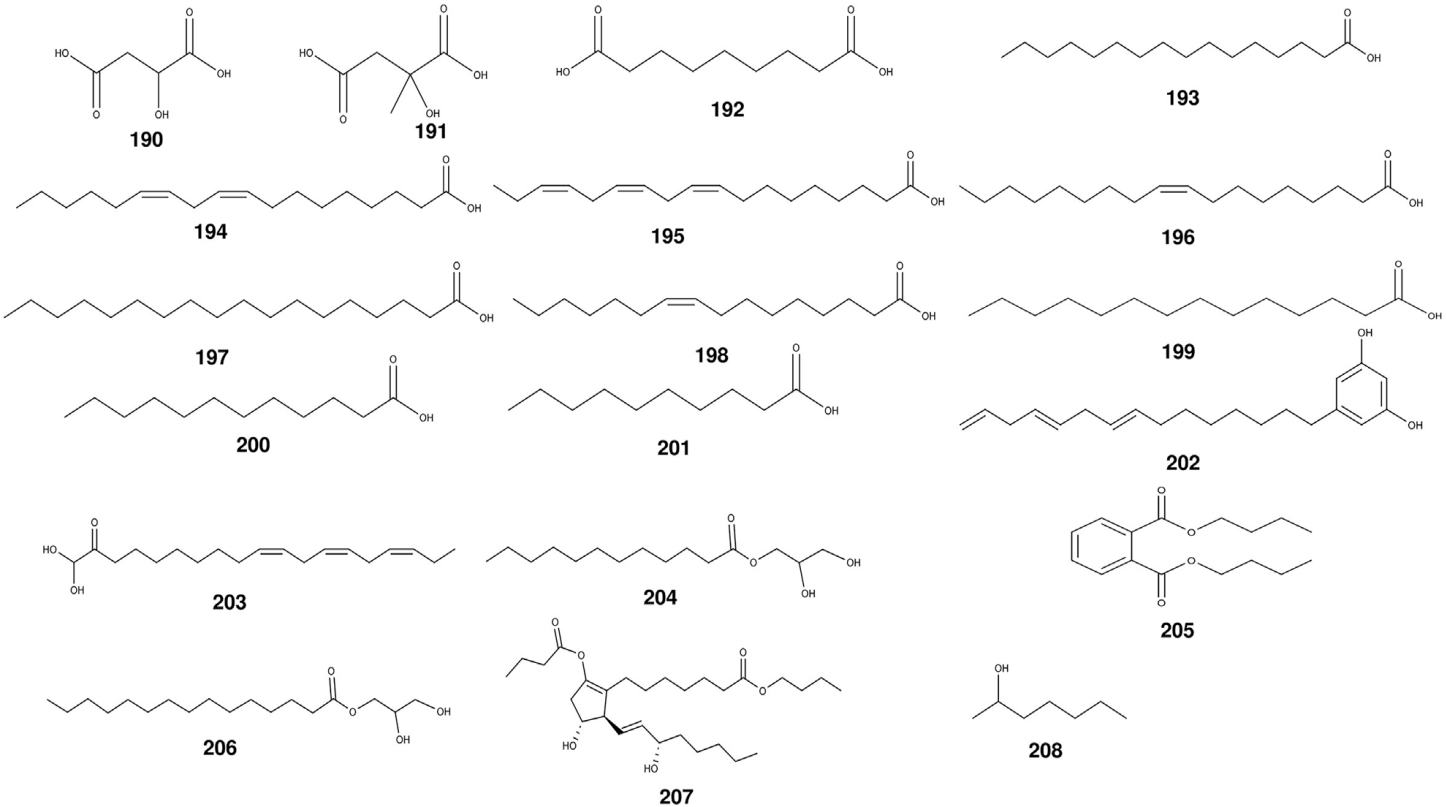
The structures of volatile oils from *S. grosvenorii*.

The seed oil of *S. grosvenorii* contains large amounts of fatty aldehydes such as fagni aldehyde, valeraldehyde, hexanal, and nonanal (Li et al., 2014). Predominantly, within the seed kernel of *S. grosvenorii*, squalene is the principal constituent of the seed oil. Concentrations of essential oils varied considerably between dried and freshly harvested fruits, with the former showing a significant increase in magnitude, ranging from about 0.2% to 0.3%, while the latter was only 0.03% (Li et al., 2014). In the fruits and roots of *S. grosvenorii*, no less than 17 fatty acids have been found (Table 4) (Figure 7). The highest contents in dried fruit essential oil were palmitic acid (n-Hexadecanoic acid) (45.609%) and linoleic acid (9, 12-octadecadienoic acid) (36.151%), conversely surpassed by butyl 2-butyrate (20.80%) and 2-heptyl alcohol (13.86%) as the prevailing constituents within the essential oil of the fresh fruit (Li et al., 2014). In the existing literature, there are very few descriptions of essential oils in *S. grosvenorii*.

**TABLE 4:** The main volatile oils from *S. grosvenorii*.

No.	Compound name	Origins	References
190	malic acid	fruit	Fang et al. (2017)
191	citramalic acid	fruit	Fang et al. (2017)
192	azelate	fruit	Fang et al. (2017)
193	palmitic acid	fruit	Fang et al. (2017)
194	linoleic acid	fruit	Zhao, 2015; Hou et al. (2020)
195	linolenic acid	fruit	Hou et al. (2020)
196	oleic acid	fruit	Zhao (2015)
197	stearic acid	fruit	Zhao, 2015; Hou et al. (2020)
198	palmitoleic acid	fruit	Zhao (2015)
199	myristic acid	fruit	Zhao (2015)
200	lauric acid	fruit	Zhao (2015)
201	capric acid	fruit	Zhao (2015)
202	5-pentadecatrienyl resorcinol	root	Zhao (2015)
203	β-(9′Z,12′Z,15′Z)-Octadecatrienoic acid monoglyceride	root	Wang (2023)
204	2,3-dihydroxypropyl (13E,15E)-octadeca-13,15-dienoate	root	Wang (2023)
205	dibutyl phthalate	root	Wang (2023)
206	Monoglycerol palmitate	root	Wang (2023)
207	butyl 2-butyrate	fruit	Li et al. (2014)
208	2-heptyl alcohol	fruit	Li et al. (2014)

### 2.5 Polysaccharides

Polysaccharides represent polymeric carbohydrate macromolecules composed of long chain monosaccharide units linked by glycosidic bonds. The content of polysaccharide was the highest in the *S. grosvenorii* flesh (7.55%) (Table 5), while the content of polysaccharide in the seed was the lowest (3.12%). The content of total sugar in *S. grosvenorii* fruit ranged from 25.17% to 38.31%, encompassing reducible saccharides accounting for values ranging from 16.11% to 32.74% (Li et al., 2014). *S. grosvenorii* polysaccharide (SGP), possessing a molecular mass of 1.93 × 10³ kDa, characterized by the constituent monomers comprising α-L-arabinose, α-L-arabinose, α-D-mannose, α-D-glucose, α-D-galactose, glucuronic acid, and galacturonic acid, configured in a stoichiometric ratio of 1:1.92:3.98:7.63:1.85:7.348 (Zhu et al., 2020). In addition, a variety of polysaccharides have been isolated and purified from *S. grosvenorii* residues, including SGPS1 (Chen et al., 2003), SGPS2 (Chen et al., 2003), SGP-1 (Gong et al., 2022), SGP-1-1 (Gong et al., 2022), SGP-2 (Gong et al., 2022), and SGP-3 (Gong et al., 2022). SGP-S1 and SGP-S2 were the first two polysaccharides obtained from *S. grosvenorii*. SGP-1, SGP-2 and SGP-3 are obtained by first dissolving SGP in distilled water, then gradient elution in distilled water, 0.05, 0.10, 0.15 and 0.20 mol/L Nacl solution, and then further concentrating and dialysis the purified fractions (Gong et al., 2022). Among them, SGP-1 has the strongest antioxidant activity (Gong et al., 2022). Furthermore, SGP-1, identified as a glucomannan derivative originating from the dried fruit of *S. grosvenorii*, is discerned by a molecular weight measuring 19.037 kDa, composed of residues including 4)-β-D-Glcp-(1→4)-, α-D-Glcp-(1→4)-, and 4)-Manp-(1 distributed in a molar proportion of 4.90:1:2.56 (Gong et al., 2022). Notably, SGP-1-1 composed of arabinose, ribose, galacturonic acid, galactose, mannose, and glucose at the molar ratio of 1.00:1.72: 2.24: 3.64: 3.89: 22.77 (Gong et al., 2021).

**TABLE 5:** The main polysaccharides from *S. grosvenorii*.

No.	Compound name	Origins	References
209	SGPS1	fruit	Chen et al. (2003)
210	SGPS2	fruit	Chen et al. (2003)
211	SGP-1	fruit	Gong et al. (2022)
212	SGP-1-1	fruit	Gong et al. (2022)
213	SGP-2	fruit	Gong et al. (2022)
214	SGP-3	fruit	Gong et al. (2022)

### 2.6 Minerals

*S. grosvenorii* is endowed with a spectrum of 19 minerals (Table 6), notably including but not limited to potassium (K), calcium (Ca), phosphorus (P), magnesium (Mg), iron (Fe), zinc (Zn), manganese (Mn), aluminum (Al), copper (Cu), lead (Pb), cadmium (Cd), and selenium (Se). Moreover, the fruit contains high levels of K, Ca, Mg and Se (Duan et al., 2023).

**TABLE 6:** The main minerals from *S. grosvenorii*.

No.	Compound name	Origins	References
215	Mn	fruit	Xia (2006)
216	Fe	fruit	Xia (2006)
217	Ni	fruit	Xia (2006)
218	Zn	fruit	Xia (2006)
219	Mg	fruit	Xia (2006)
220	Ca	fruit	Xia (2006)
221	Pb	fruit	Xia (2006)
222	Cu	fruit	Xia (2006)
223	K	fruit	Xia (2006)
224	Na	fruit	Xia (2006)
225	Cd	fruit	Xia (2006)
226	Sr	fruit	Xia (2006)
227	Ba	fruit	Xia (2006)
228	Cr	fruit	Xia (2006)
229	Al	fruit	Xia (2006)
230	Be	fruit	Xia (2006)
231	Ti	fruit	Xia (2006)
232	P	fruit	Lei (2017)
233	S	fruit	Lei (2017)

### 2.7 Vitamin

*S. grosvenorii*, there are also some vitamins such as Thiamine (Vitamin B1), Riboflavin (Vitamin B2), and Ascrobic acid (Vitamin C) (Table 7). Among them, the content of Vitamin B1 in every 100 g of fresh fruit is 338 mg, and Vitamin B2 is 123 mg. The amount of vitamin C detected in *S. grosvenorii* varies greatly depending on the variety, form, source, growth stage, maturity, differences in drying equipment, and process conditions used by suppliers. Remarkably, this concentration exceed that discerned within botanical counterparts such as citrus, apple, pear, grape, and persimmon. The content of vitamin C in *S. grosvenorii* fresh fruit (ranging between 339 and 461 mg per 100g) outpaces its dried fruit (ranging between 24.6 and 38.7 mg per 100g) by a factor exceeding tenfold (Ji, 2016). Vitamin C tends to degrade at high temperatures. Therefore, in order to minimize damage to the inherent vitamin C of LHG, it is preferable to preserve LHG under low-temperature vacuum drying conditions (Ji, 2016). In addition, a new strategy to protect LHG, is through the use of microwave heating. The technique involves applying microwave power of 80 W, with a time parameter of 30 min and a microwave interval ratio of 1:1 (Zhou et al., 2018b). This method not only extends the storage life of LHG, but also minimizes the loss of water and vitamin C content.

**TABLE 7:** The main vitamins from *S. grosvenorii*.

No.	Compound name	Origins	References
234	Vitamin B1	fruit	Thakur et al. (2023)
235	Vitamin B2	fruit	Thakur et al. (2023)
236	Vitamin C	fruit	Zhou et al. (2018b)
237	Vitamin E	fruit	Zhang et al. (2020b)

### 2.8 Others

*S. grosvenorii* and its root, stem, and leaf also contains wooden fat element, phenolic acids, anthraquinones, alkaloids, sterols, fatty acid, furfural, aromatic, diterpene lactone, hine ring, brain glycosides, and ceramide compounds (Wu et al., 2023).

## 3 Pharmacological effects

*S. grosvenorii* has been extensively documented in various traditional Chinese medicine references such as *The Dictionary of Traditional Chinese Medicine, National Compendium of Materia Medica, Lingnan Materia Medica, Color Atlas of Chinese Herbal Medicine, Chinese Herbal Medicine and Chinese Pharmacopoeia*. These references highlight the established role of *S. grosvenorii* in traditional Chinese medicine for removing lung heat, resolving phlegm turbidity, addressing paramesoteric swelling, mitigating cough, alleviating sore throat, addressing hoarseness, chronic laryngitis, chronic bronchitis, and other ailments afflicting the respiratory system (Ji, 2016). Traditionally, traditional Chinese medicine believes that *S. grosvenorii* is cool in nature, sweet in taste, and meridian distribution in the lung and large intestine (2005).

Given the wide range of documented medicinal and food values of *S. grosvenorii*, increasing attention has been given to studying its pharmacological effects and the mechanisms of diseases treatment. In this paper, the pharmacological effects of *S. grosvenorii* are summarized as follows (Table 8).

**TABLE 8:** The pharmacological effects of *S. grosvenorii*.

Pharmacological effects	Detail	Extracts/Compounds	Concentration/Dose	*In Vivo* */* *In Vitro*	Ref.
Cough Suppressant & Expectorant	Reduced the frequency of cough induced by ammonia water and prolong the latency of cough induced by concentrated ammonia water in mice	①*S. grosvenorii* decoction; ②Mog V isolated by washing with 50% alcohol of *S. grosvenorii*	①50 g/kg; ②300 mg/kg	*In vivo*	Liu et al. (2007)
	Reduced the number of cough episodes caused by citrate or capsaicin, or mechanical stimulation in guinea pig, and prolonged the cough incubation period in guinea pig	the aqueous extract of *S. grosvenorii*	0.02, 2.3, 4.6, 9.2 g/kg	*In vivo*	Li et al. (2008)
Bacteriostasis and caries prevention	Alleviated POST by significantly inhibiting the growth of *S. mutans*	SGEs	10 mg/mL	*In vitro*	Zhou et al. (2022b)
	Inhibited the grown of *S. mutans/A. actinomycetemcomitans/F. nucleatum/C. albican*	β-amyrin, aloe emodin, aloe-emodin acetate, 5α,8α-epidioxy-24(R)-methylcholesta-6,22-dien-3β-ol and p-hydroxyl benzyl acid isolated from the leaves of *S. grosvenorii*	100, 50, 25, 12.5, 6.25 and 3.125 μg/mL	*In vitro*	Zheng et al. (2011)
	The cariogenic effect on the molar teeth of SD rats was lower than sucrose	*S. Grosvenorii* juice concentrate and Mog	2% (w/v)	*In vivo*	Yan (2009)
	An appropriate proportion of the *S. grosvenorii, Platycodon grandiflorum, Chaenomeles sinensis Koehne* mixture had an effect on pathogenic oral bacteria but not on non-pathogenic oral bacteria	SGEs	0.01%–0.5%	*In vitro*	Lee et al. (2021)
Anti-inflammation (allergic/non-allergic)	Alleviated pulmonary inflammatory infiltration in OVA -induced asthmatic mice	①Mog V; ②SGEs; ③11-O-MogV	①50 mg/kg (purity≥95%); 2, 5, or 10 mg/kg (purity≥95%); ②——; ③——	*In vivo*	(Jia et al., 2019; Song et al., 2019; Liu et al., 2021b)
	Inhibiting Th2 and Th17 cytokines and increasing Th1 cytokines to inhibit asthma in mice	SGEs	200 and 100 mg/kg	*In vivo*	Sung et al. (2019)
	Decreased the number of activated T cells, B cells, and neutrophils in BALF, lung tissue, mesenteric lymph nodes, and peripheral blood mononuclear cells; reduced the secretion of cytokines in BALF and downregulated the expression of proinflammatory cytokines MUC5AC, transient receptor potential vanilloid receptor 1 and transient receptor potential ankyrin 1 in COPD mice’s lung tissue	SGEs	*In vitro*: 50, 100, 200 μg/mL; *In vivo*: 25, 50, 100 mg/kg	*In vivo* and *in vitro*	Kim et al. (2023)
	Interfered with the activation of PI3K/AKT/IKK/MAPK pathway and NF-κB pathway to downregulate iNOS and COX-2 gene expression in macrophages	SGEs	600 or 800 μg/mL	*In vitro*	Pan et al. (2009)
	Regulated immune dysfunction and address skin barrier abnormalities to improve allergic skin inflammation	SGREs	200 or 400 mg/kg/d	*In vivo*	Sung et al. (2020)
	Activated of MAPK pathway to inhibit the expression of inflammatory mediators iNOS, cox-2, 5-LOX, PGE2, LTB4, and cytokines IL-1β, IL-6, TNF-α, and downregulate MMP-1, -2, -9 and -13, thereby reducing inflammation; relieved the discomfort of OA - induced	SGREs	150 or 200 mg/kg	*In vivo*	Lee et al. (2023)
	Inhibited of oxidative stress, apoptotic cascade, inflammatory processes, precancerous lesions and fibrosis progression in the liver of NASH rats	SGREs	Concentrations above 0.2%	*In vivo*	Uno et al. (2023)
	Reduced the inflammatory cascade and oxidative stress to reduce intestinal damage caused by heat stress	MGEs	600 mg/kg	*In vivo*	Lu et al. (2023b)
	Blocked NF-κB signaling pathway, reducing inflammatory cell infiltration, proinflammatory cytokine production, lung wet/dry weight ratio, MPO activity	Mog V	5 and 10 mg/kg	*In vivo*	Shi et al. (2014)
	Attenuated the activation of the TLR4-MyD88 cascade and the AMPK/AkT-nuclear factor E2-associated Factor 2 (Nrf2) signaling axis, thereby reducing LPS-induced neurotoxicity and inflammation	Mog V	*In vitro* *:* 3.125, 6.25, 12.5, 25, and 50 μM*;* 6.25, 12.5, and 25 μM	*In vitro*	Liu et al. (2021c)
	Inhibited of IL-9/IL-9 receptor pathway attenuates the activities of trypsin and cathepsin B in AR42J and primary acinar cells induced by cerulein and LPS in tandem	Mog IIE	*In vivo* *:* 10 mg/kg; *in vitro* *:* 5, 10, and 20 μM	*In vivo* and *in vitro*	Xiao et al. (2020)
	Downregulated the mRNA and protein levels of TLR4 and its downstream NF-κB p65 to reduce the inflammation induced by diabetic nephropathy	SGP-1-1	50, 100 and 200 mg/kg/d	*In vivo*	Gong et al. (2021)
Anti-aging and anti-oxidative stress Anti-aging and anti-oxidative stress	Increased SOD activity and decreased MDA content in brain tissue and serum of aging mice induced by D-galactose	Mog	1.5, 0.75, 0.38 g/kg	*In vivo*	Xiao et al. (2014)
	Inhibited the oxidation of lipid and protein in meat under vacuum storage conditions	MGEs	7, 10 and 15 g MGEs per 100 g mixture	*In vitro*	Cheng et al. (2017)
	Prolonged the average and maximum lifespan of *Drosophila*	Mog	20%, 10%, 5%	*In vivo*	Xiao et al. (2014)
	Scavenged hydroxyl radical and superoxide anion radical, inhibited the production of MDA during erythrocyte autooxidation hemolysis and hepatic lipid peroxidation in rats	Mog, Mog V	Hydroxyl radical scavenging assay: 10, 30, 50, 70, 100 μg/mL; superoxide anion free radical scavenging assay: 0.25, 0.44, 0.74, 1.47, 2.21, 2.94 mg/mL (Mog); 0.029, 0.059, 0.118, 0.176, 0.235, 0.294 mg/mL (Mog V); autooxidative hemolysis of erythrocytes: 1.15, 0.81, 0.46, 0.12, 0.14 mg/mL (Mog); MDA assays: 1.25, 0.875, 0.5, 0.125, 0.042 mg/mL (Mog)	*In vivo* and *in vitro*	Qi et al. (2006)
	Enhanced mitochondrial biosynthesis and reduce intracellular ROS accumulation to promote first polar body extrusion, thereby promoting oocyte maturation	Mog V	20 μM	*In vitro*	Nie et al. (2020)
	Upregulated SIRT1 to reduce oxidative stress, thereby reducing ROS, spindle formation, and chromosomal abnormalities	Mog V	50 μM	*In vitro*	Nie et al. (2019)
	Dose dependent to cut the level of ROS in the PC12 cells, level of DPPH free radical, apoptosis and necrosis cells	SGP	20, 200, 2000 μg/mL	*In vitro*	Zhu et al. (2020)
	Reduced the oxidative stress caused by diabetic nephropathy by down-regulating the activation of TLR4/NF-κB p65 pathway; stimulated the production of SOD and reducing the production of MDA	SGP-1-1	50, 100, 200 mg/kg/d	*In vivo*	Gong et al. (2021)
Reduce blood sugar	Decreased blood glucose, TC, TG and hepatic MAD levels; increased HDL-C levels and hepatic antioxidant enzyme activities in alloxan-induced diabetic mice	MGEs	100, 300, 500 mg/kg	*In vivo*	Qi et al. (2008)
	Dose-dependently reduced FBG, GSP, serum insulin, HOMA-IR and serum atherogenic lipid profile in diabetic mice	MGEs	150, 300 mg/kg	*In vivo*	Liu et al. (2019)
	Inhibited the activation of HO-1 to play an antioxidant role in diabetic nephrotic mice	Mog	150, 300 mg/kg	*In vivo*	Song et al. (2007)
	Increased insulin secretion in the fasting state, enhanced kidney function, and enhanced antioxidant properties of liver and plasma to treat spontaneous diabetes in Goto-Kakizaki rats	SG-ex	Mog V, 11-O-Mog V, Mog IV, Mog III and siamenoside I were approximately 2.1, 0.2, 0.8, 0.7 and 0.3%, respectively	*In vivo*	Suzuki et al. (2007)
	Inhibited wheat glycoenzyme activity plays an antihyperglycemic role in rats	SG-ex, the triterpene glycoside concentrate from SG-ex (SG-gly)	0.1 g/kg	*In vivo*	Suzuki et al. (2005)
	Decreased FBG levels, improved insulin resistance, increased GLP-1 expression, and activated hepatic AMPK.	L-SGgly	L-SGgly was isolated from 50% ethanol extract, containing 11-oxo-mog V, Mog V, Mog III, Mog IIE, Mog IIIA1, Mog IIA1, and Mog IA1, with a total Mog content of 54.4%, with Mog IIA1 and Mog IA1 containing 15.7% and 12.6%, respectively	*In vivo*	Zhang et al. (2020a)
Inhibit lipid deposition and obesity	Improved hepatocyte polymorphism, lipid accumulation and steatosis in diabetic mice	MGEs	300 mg/kg	*In vivo*	Liu et al. (2019)
	Activated AMPK pathway and regulated the expression of SREBP1, PPAR-c and PPAR-α can improve the imbalance between lipid acquisition and lipid clearance, thereby protecting mice against hepatic steatosis induced by high-fat diet	Mog V	*In vivo*: 25, 50, and 100 mg/kg/d; *In vitro*:15, 30, 60, 120 μM	*In vivo* and *in vitro*	Li et al. (2020)
	Enhanced the diversity of intestinal flora and changed its composition, reduced the degradation of liver lysine metabolism and inhibited fatty acid β-oxidation	NSG	——	*In vivo*	Wang et al. (2023)
Anti-depression-like effect	Reduced the damage of corticosterone to PC12 cells; inhibited inflammation and oxidative stress-related pathways and BDNF/TrkB/AKT pathway exerted antidepressant effects	Mog V	*In vitro*: 0, 30, or 90 μM; *In vivo*: 10 or 30 mg/kg/d	*In vivo* *and* *in vitro*	Liu et al. (2023a)
Anti-fatigue effects	Enhanced exercise capacity, hypoxia and heat tolerance, and reduced exercise-induced fatigue in Kunming mice in a dose-dependent manner	SGEs	9, 11, 13, 15 (the dose that works best), 17 g/kg/d	*In vivo*	Yao et al. (2007)
	Improved the swimming ability of mice, increased glycogen stores in liver and muscle, and reduced the reduction of blood lactate and serum urea nitrogen concentration	SGEs	100, 200, 400 mg/kg	*In vivo*	Liu et al. (2013a)
	Increase in rat skeletal muscle fatigue p-AMPK alpha, AMPK alpha, pgc-1 alpha and expression of TFAM to play a role of fatigue	Mogrol	20, 40, 80 mg/kg	*In vivo*	Guo and Wen (2022)
	Enhanced blood perfusion and oxygen transport capacity in muscle tissue to enhance the exercise capacity of male SD rats	Flavonoids	100, 200, 400 mg/kg	*In vivo*	Chen et al. (2020)
	Improved the activity of antioxidant enzymes; removed excess free radicals produced by exhaustive exercise; inhibited lipid peroxidation, protect myocardial mitochondria; inhibited the apoptotic process	Leaf-derived flavonoids	100, 200, 400 mg/kg	*In vivo*	(Guo and Peng, 2012; Xie, 2018)
	Accelerated glycogen storage and lactic acid metabolism to enhance mice’s ability to resist fatigue and hypoxia tolerance	Mog	75, 150, 300 mg/kg	*In vivo*	Xia et al. (2012)
Anti-schizophrenia	Alleviated PPI deficits and social withdrawal induced by MK801	Mog V	50 mg/kg	*In vivo*	(Ju et al., 2015; Ju et al., 2020)
	Reduced total movement distance and number of spontaneous movements in MK801-induced schizophrenia mice; enhanced cellular and neurochemical responses to MK801 within mPFC	Mog V	50 mg/kg	*In vivo*	Ju et al. (2015)
	Promoted neurite outgrowth, inhibited cell apoptosis, and inhibited [Ca^2+^]i release	Mog V; 11-oxo-mogrol	2.5, 5 mg/mL (Mog V); 0.1 μM (11-O-Mog)	*In vivo*	Ju et al. (2020)
	Reversed MK801-induced AKT inactivation and mTOR phosphorylation to modulate schizophrenia	11-oxo-mogrol	0.1 μM	*In vivo*	Ju et al. (2020)
Anti-Parkinson’s disease	Alleviated MPP^+^-induced SY5Y cell damage	Mog V, mogrol	10, 50, 100 μM	*In vitro*	Tang (2021)
	By adjusting the synthesis of amino acids, lipid metabolism and intestinal - brain shaft steady state so as to reduce the mitochondrial dysfunction and the substantia nigra neurons lost, to improve movement function defects of MPTP induced PD mice and the pathological changes of the brain	Mog V	10, 30 mg/kg	*In vivo*	Tang (2021)
	Reduced ROS overproduction, restored mitochondrial membrane potential, increased oxygen consumption and ATP production, and reduced the decrease in the number of apoptotic cells reversed rotenone-induced motor deficits and dopaminergic neuronal damage in PD mice in a dose-dependent manner	Mog V	10 mg/kg	*In vivo*	Ju et al. (2020)
	Downregulated the acetylation of SIRT3 in nigra and SH-SY5Y cell lines; interfereed with the activity of SOD2, thereby enhancing its neuroprotective effect	Mog V	*In vivo*: 10 mg/kg; *In vitro*: Mog V25, Mog V50, and Mog V100 treated with 25 μM, 50 μM, and 100 μM MV doses, respectively	*In vivo* *and* *in vitro*	Luo et al. (2022)
Anti-fibrosis	Without altering the fatty degeneration inhibiting fibrosis to prevent CDAA - HF - T (-) -induced NASH mice	SGEs	0.2%, 0.6%, 2% SGEs (mogroside compounds combined with Mog V, 11-O-Mog V, Mog IV, and Siamenoside I was 69%)	*In vivo*	Suzuki-Kemuriyama et al. (2021)
	Prevented liver fibrosis by preventing the NF-κB pathway from being active	SGEs	0.06, 0.2, 0.6, 2.0, and 6.0% SGEs in drinking water	*In vivo*	Uno et al. (2023)
	Regulated toll-like receptor 4 (TLR4)/MyD88 - MAPK signal cascade to reduce lung inflammation and suppress the ECM deposition	Mog IIIE	*In vivo*: 1, 10, 20 mg/kg; *In vitro*: 10 μM	*In vivo* *and* *in vitro*	Tao et al. (2017)
	Inhibited the expression of TLR4/MyD88/NF-κB signaling pathway to release inflammatory cytokines, thereby reducing myocardial fibrosis	Mog IIIE	*In vivo*: 1, 10 mg/kg; *In vitro*: 100, and 200 μmol/L	*In vivo* *and* *in vitro*	Yanan et al. (2023)
	Reduces lung inflammation by activating AMPK. Inhibits TGF-β1-induced mesenchymal transformation of alveolar epithelial cells and transdifferentiation of myofibroblasts through activation of the TGF-β1/Smad2/3 signaling pathway	Mogrol	*In vivo*: 1, 5, 10 mg/kg; *In vitro*: 1, 5, 10 μM	*In vivo* *and* *in vitro*	Liu et al. (2021a)
Anti-cancer	By attenuating TPA-induced activation of ERK1/2, MAPK, JNK1/2, PI3K and Akt, nuclear translocation of NF-κB subunit and phosphorylation of IkBa and p65 are inhibited, thereby inhibiting 7, 12-DMPA-TPA-induced skin tumorigenesis	SGEs	2.5 or 10 mg in 200 mL acetone	*In vivo*	Weerawatanakorn et al. (2014)
	Reduced the viability of PC-3 and T24, induced G1 phase cell cycle arrest, ER stress and apoptosis, thereby treating prostate and bladder cancer	LLE, Mog	3 μg/mL (LLE); 2000 μg/mL (Mog)	*In vitro*	Haung et al. (2023)
	Inhibited the second-stage carcinogenesis of ONOO2/TPA-induced skin tumors in mice	Mog V; 11-O-Mog V	0.0025%, 2.5 mg/100 mL in drinking water	*In vivo*	Takasaki et al. (2003)
	Inhibited the second-stage carcinogenesis of DMBA/TPA-induced skin tumors in mice	11-O-Mog V	0.0025%, 2.5 mg/100 mL in drinking water	*In vivo*	Takasaki et al. (2003)
	Inhibited TPA-induced production of EBV-EA in Raji cells	karounidiol dibenzoate, karounidiol 3-benzoate, isomultifiorenol, β-amyrin; 10α-cucurbitadienol; mogrol; 5α,6α-epoxymogroside lE1; 11-oxomogroside lA1; 11-oxomogroside lE1; Mog I A1; Mog IE1; Mog I E; Mog llI; SiamenosideI; Mog IVA; Mog IV E; 11-oxo-Mog V; Mog V; 11-oxomogrol; β-carotene	1000, 500, 100, 10 mol ratio/TPA	*In vivo*	Ukiya et al. (2002)
	Inhibited the proliferation of human prostate cancer cell line DU145, human liver cancer cell line HepG2, human lung cancer cell line A549, human nasopharyngeal carcinoma cell line CNE1 and CNE2	Mogrol	25, 100, 200 μmol/L	*In vitro*	Fu et al. (2016)
	Induced apoptosis and block G0/G1 phase, thereby inhibiting the proliferation of tumor cells and playing an anti-tumor role	Mogrol	①25, 100, 200 μmol/L; ②0.1, 1, 10, 100, 200, 250 μmol/L	*In vitro*	(Liu et al., 2015; Fu et al., 2016)

### 3.1 Cough suppressant & expectorant

Cough is a sudden, often involuntary discharge of air from the lungs with a characteristic, easily recognizable sound (Farzan, 2008). It has the function of defending against harmful substances in the respiratory tract and maintaining airway patency by clearing excessive airway secretions. Expectoration or sputum refers to the act of coughing and ejecting substances produced in the respiratory tract (Farzan, 2008).

*S. grosvenorii* has been used in southern China for more than 300 years as a herbal medicine to clear the throat and moisten the throat. There was a screening experiments for the anti-tussive active ingredients of *S. grosvenorii* have been carried out. Liu et al. conducted an experiment in which they concentrated the crude powder of *S. grosvenorii* to a concentration of 1.5 g/mL of raw drug. The raw drug was then extracted with water and the resulting filtrate was eluted with water, 50% ethanol, and 95% ethanol on AB-8 macroporous adsorbent resin. This process yielded three parts of *S. grosvenorii:* a water-soluble part, a 50% ethanol-soluble part, and a 95% ethanol-soluble part (Liu et al., 2007). The 50% ethanol-soluble part was further purified by D-280 anion exchange resin to remove alcoholic flavor, and the resulting effluent was concentrated to obtain the Mog crude product (Liu et al., 2007). After repeated purification on an Octadecylsilyl column, the purity of Mog V was found to be over 94% (Liu et al., 2007). In a mice coughing experiment conducted using *S. grosvenorii* decoction, it was found that the herb had a certain cough-suppressing effect, but a larger dose (50 g/kg of raw drug) was required to achieve this effect. However, when Mog V (75 mg/kg) was isolated from the effective extracted part (50% alcohol washed) of *S. grosvenorii*, it showed a significant antitussive effect, with an inhibition rate of 52.93%, indicating a certain quantitative effect relationship (Liu et al., 2007). Another experiment involving phenol red excretion in mice showed that while 50 g/kg of *S. grosvenorii* raw drug did not have a significant expectorant effect, Mog V at a dose of 150 mg/kg significantly increased phenol red excretion in mice (Liu et al., 2007). Another experiment using guinea pigs, which have a more similar cough reflex to humans, showed that the aqueous extract of *S. grosvenorii* significantly reduced the number of cough episodes or prolonged cough latency induced by citrate or capsaicin, and inhibited cough induced by mechanical stimulation in guinea pigs (Li et al., 2008). This again indicates the antitussive effect of *S. grosvenorii*.

The above experimental results indicate that Mog V is the main active component of *S. grosvenorii,* and the activity of the monomer after isolation and purification is more potent than that of the total aqueous decoction.

### 3.2 Bacteriostasis and caries prevention

Postoperative sore throat (POST), constitutes a prevalent sequel following tracheal intubation and subsequent extubation conducted within the milieu of general anesthesia (El-Boghdadly et al., 2016). The underpinning etiological factor resides within the constrained immunological competence exhibited by the patient throughout the surgical procedure. This physiological limitation manifests as a propitious milieu for the atypical proliferation of oropharyngeal bacterial consortia. Consequent to this microbial overgrowth, deleterious sequelae ensue, comprising injurious ramifications for the oral mucosal integrity, culminating in ulcerative lesions and acute pharyngitis (Scuderi, 2010). It has been showed that adding 10 mg/mL *S. grosvenorii* extracts (SGEs) to brain heart infusion-sheep blood agar substrate alleviates POST by significantly inhibiting the growth of *Streptococcus mutans* (*S. mutans*) (Zhou et al., 2022b). The Glucosyltransferase (GTF) enzyme derived from *S. mutans* constitutes a pivotal virulence determinant in the etiology of dental caries. β-amyrin, aloe emodin, aloe-emodin acetate, 5α,8α-epidioxy-24(R)-methylcholesta-6,22-dien-3β-ol and *p*-hydroxyl benzyl acid isolated from the leaves of *S. grosvenorii*, have demonstrated notable antibacterial efficacy (Zheng et al., 2011). Notably, among them, aloe emodin has strongest antibacterial effect against growth of *S. mutans*, *A. actinomycetemcomitans, F. nucleatum and C. albicans* (Zheng et al., 2011). Conversely, the derivative aloe-emodin acetate exhibited diminished inhibitory effects on *S. mutans, A. actinomycetemcomitans, and C. albicans* when compared to aloe emodin, and exhibited no growth suppression of *F. nucleatum* (Zheng et al., 2011). *p*-Hydroxyl benzyl acid demonstrated pronounced inhibition against *S. mutans, F. nucleatum and C. albicans*, but did not influence *A. actinomycetemcomitans* (Zheng et al., 2011). 5,8-epidioxy-24(R)methylcholesta-6,22-dien-3-ol had moderate activities against *S. mutans, A. actinomycetemcomitans, F. nucleatum* and *C. albicans* (Zheng et al., 2011). β-amyrin exhibited only marginal inhibition against *S. mutans* and *F. nucleatum*, whereas aloe emodin demonstrated moderate activities analogous to aloe-emodin acetate against C. albicans. Notably, aloe emodin exhibited significantly more potent antibacterial action against *S. mutans, A. actinomycetemcomitans*, and *F. nucleatum* when juxtaposed with aloe-emodin acetate (Zheng et al., 2011).

Caries and periodontitis stand as emblematic oral diseases in humans (Jorn et al., 2005). The main cariogenic bacteria in the oral cavity are *S. mutans* (Hamada et al., 1984). The pathogenic cascade orchestrated by *S. mutans* in caries development involves a tripartite sequence. Firstly, the bacterium adheres to the acquired dental membrane in a sucrose independent manner. Subsequently, adhesion to tooth surfaces, contingent upon sucrose, is facilitated through GTF mediation. Lastly, the substrate is metabolized to produce acid (Daboor et al., 2015). Conspicuous investigations have illuminated an intriguing facet of *S. grosvenorii*, namely its role as a natural sweetener with diminished cariogenic potential. Experimental endeavors conducted *in vitro* have distinctly portrayed the subdued growth rate and adherence of *S. mutans* in culture mediums enriched with Mogs, in stark contrast to their sucrose-rich counterparts. The culture mediums abundant in Mogs have been observed to markedly curtail the proliferation and propagation of *S. mutans*. Further empirical inquiries conducted *in vivo* have unequivocally indicated a diminished cariogenic impact of concentrated *S. grosvenorii* juice upon the molars of SD rats when compared with sucrose (Yan, 2009).

While mouthwashes have been harnessed to deter the adhesion of oral microorganisms onto dental biofilms and tooth surfaces, concurrently manifesting antibacterial attributes to thwart the inception of dental complications, synthetic mouthwash formulations encompass an amalgam of compounds preservatives, artificial colors, and taste correction agents. Long-term use of such chemical-laden mouthwashes could lead to gustatory disorders, discoloration of oral hard and soft tissues, allergic reactions, and even oral carcinoma (McCullough and Farah, 2008). Furthermore, the prolonged utilization of chemical mouthwashes might potentially precipitate the eradication of oral microbial communities, thus impinging upon the nitrate-nitrite-NO pathway within salivary secretions, potentially engendering the emergence of cardiovascular maladies and septicemia (Blot, 2021). Remarkably, studies have found that *S. grosvenorii* alone demonstrated no discernible impact on 11 pathogens in the oral cavity, the natural extract mixture of *S. grosvenorii*, *Platycodon grandiflorum, Chaenomeles sinensis Koehne*, enzyme salt, xylitol, mint, green tea, lemon, propolis, silicon dioxide and magnesium stearate exhibited notable anti-inflammatory attributes, evinced by its capacity to inhibit lipopolysaccharide (LPS)-induced NO production and foster the viability of RAW 264.7 cells (Lee et al., 2021). More importantly, natural extracts mixed in proper proportions had an effect on pathogenic oral bacteria but not on non-pathogenic oral bacteria (Lee et al., 2021).

Collectively, these findings indicate that both the fruits and leaves of *S. grosvenorii* hold promise in terms of their antimicrobial properties. Additionally, various active compounds isolated from the leaves exhibit inhibitory effects on different bacterial species. Notably, Mogs demonstrate preventive efficacy against tooth decay. Combining natural extracts from *S. grosvenorii* with other natural extracts in appropriate proportions has a good prospect of replacing traditional chemical-based mouthwash formulations, and is worthy of further exploration and consideration.

### 3.3 Anti-inflammation (allergic/non-allergic)

In recent years, the anti-inflammatory effects of *S. grosvenorii* have been supported by a large number of experimental data. So far, it has been confirmed that *S. grosvenorii* has therapeutic effects on the inflammation of the lungs, kidneys, skin, intestines, bones and joints, liver, pancreas and nervous system.

Several studies have provided evidence of the effectiveness of SGEs in reducing lung inflammation. Experiments conducted on both *in vivo* models of lung inflammation induced by LPS have shown that treatment with SGEs significantly affects the activation of mitogen-activated protein kinase (MAPK)—nuclear factor-κB (NF-κB) in human bronchial epithelial cell line (BEAS-2B) when exposed to LPS. Consequently, this modulation inhibits the production of inflammatory mediators (Kim et al., 2023). Moreover, in a mouse model of chronic obstructive pulmonary disease (COPD) induced by cigarette smoke extract (CSE) and LPS, SGEs have been found to decrease the number of activated T cells, B cells, and neutrophils, as well as reduce inflammatory factor infiltration in various areas like bronchoalveolar lavage fluid (BALF), lung tissue, mesenteric lymph nodes, and peripheral blood mononuclear cells. Furthermore, SGEs downregulate the expression levels of proinflammatory cytokine MUC5AC, transient receptor potential vanilla-like receptor 1, and transient receptor potential fixing protein 1 in lung tissue (Kim et al., 2023). The anti-inflammatory mechanism of SGEs appears to involve inhibiting the activation of NF-κB by interfering with the activation of inosine phosphate 3 kinase (PI3K)/protein kinase B (Akt)/kappa B kinase inhibitor (IKK) and MAPK pathway (Pan et al., 2009). Ultimately, this effect leads to a downregulation of the expression of inflammatory inducible nitric oxide synthase (iNOS) and cyclooxygenase-2 (COX-2) genes in macrophages (Pan et al., 2009).

Studies have validated the effectiveness of *S. grosvenorii* residue extracts (SGREs) in addressing lung inflammation, skin inflammation, osteoarthritis (OA), and nonalcoholic steatohepatitis (NASH). SGREs have been found to suppress the expression of Th2 and Th17 cytokines while boosting the levels of Th1 cytokines (Kim et al., 2023). In a NC/Nga murine model treated with *Dermatophagoides farinae* mite antigen extract, SGREs (comprising grosvenorine, kaempferitrin, and Mogs) at doses of 200 and 400 mg/kg exhibited superior improvement in allergic skin inflammation compared to 5 mg/kg dexamethasone. This effect was achieved through the regulation of immune dysfunction and resolution of skin barrier abnormalities (Sung et al., 2020). SGREs exhibit potential as a bioactive treatment for OA. They not only help improve weight distribution on the back foot, thereby reducing discomfort associated with OA, but also have the ability to inhibit various inflammatory mediators such as iNOS, COX-2, 5-lipoxygenase (5-LOX), prostaglandin E2 (PGE2), and leukotriene B4 (LTB4) through the activation of the MAPK/NF-kB pathway. Additionally, they can impact the expression of inflammatory cytokines (including interleukin (IL)-1β, IL-6, tumor necrosis factor (TNF)-α) and cartilage-degrading enzymes (including matrix metalloproteinases (MMP)-1, -2, -9, and -13) (Lee et al., 2023). In a male Hsd: SD rat model of choline-deficient, methionine-deficient, L-amino acid-defined (CDAA)-induced NASH, oral concentrations of SGREs greater than 0.2% regulate the NF-κB pathway by regulating CD44 expression. At the same time, oxidative stress, apoptotic cascade, inflammatory processes, precancerous lesions, and fibrotic progression in the rat liver environment were also inhibited, again demonstrating the inherent anti-inflammation and antioxidant properties of SGREs (Uno et al., 2023).

Mogroside-rich extracts (MGEs) exhibits the potential to mitigate intestinal impairment resulting from thermal stress by ameliorating the cascades of inflammation and oxidative stress, as indicated by previous investigations (Lu et al., 2023b). This finding suggests that MGEs may have the potential to mitigate the consequences of increased thermal stress associated with global climate change.

Mog V has been shown to reduce lung and neuroinflammation. A study established an affinity chromatography strategy to evaluate the anti-asthmatic effects of SGEs using immobilized toxomatine 3 acetylcholine receptor (M3R). The results showed that the flavor components in SGEs that bind to this receptor are Mog V and 11-O-Mog V. The anti-asthmatic effects of these ingredients when used in concert were more significant than SGEs alone (Jia et al., 2019). These findings collectively underscore the promise of Mog V and 11-O-Mog V as candidates for functional food additives. Subsequent studies revealed 93 pathways associated with ovalbumin (OVA)-induced pneumonia through transcriptomic and proteomic analysis. Notably, Mog V effectively inhibited the activation of NF-κB and JAK-STAT pathways, subsequently inhibiting Igha, Ighg1, NF-κB, Jak1 and Stat1 activation to exert discernible anti-inflammatory effects (Song et al., 2019; Dou et al., 2022). A metabolomic analysis was performed using liquid chromatography-mass spectrometry (LC-MS) to elucidate the regulatory role of Mog V in a mouse model of asthma. The results showed that Mog V is involved in regulating six key pathways in asthmatic mice, encompassing vitamin B6 metabolism, taurine and hypotaurine metabolism, ascorbic acid and aldarate metabolism, histidine metabolism, pentose and glucuronic acid interconversion, and the citric acid cycle (TCA cycle). By conducting these experiments, it was observed that the administration of 50 mg/kg Mog V demonstrated similar effects to the positive control, 50 mg/kg Suhuang Zhike Jiaonang, in reducing the presence of biochemical factors and pulmonary inflammatory infiltration in mice with OVA-induced asthma (Liu et al., 2021b). In addition, Mog V was shown to relieve inflammation in a mouse model of LPS-induced acute lung injury. Pretreatment with Mog V at doses of 5 or 10 mg/kg resulted in the attenuation of inflammatory cell invasion, reduction in pro-inflammatory cytokine production, decreased lung wet/dry weight ratio, inhibition of myeloperoxidase activity, and mitigation of lung histopathological changes. These effects were achieved through the blocking of the NF-κB signaling pathway (Shi et al., 2014). In addition, Mog V demonstrated a suppressive effect on the expression of iNOS and COX-2 (Shi et al., 2014). However, Mog V was slightly less effective than dexamethasone (2 mg/kg) (Shi et al., 2014). Mog V also has a lessening effect on neuroinflammation. One evidence is that Mog V exhibits the potential to attenuate the activation of the TLR4-MyD88 cascade and the AMPK/AKT-nuclear factor erythroid2-related factor 2 (Nrf2) signaling axis, thereby mitigating the produce of proinflammatory mediators (namely, TNF-α and IL-1β) within microglial populations, the innate immune effector cells of the central nervous system (CNS). In addition, the secretion of IL-18, IL-6, COX-2, iNOS and high mobility group box 1 (HMGB1) was also inhibited by Mog V, thereby inhibiting LPS neurotoxicity (Liu et al., 2021c).

Mog IIE is one of the main bioactive components in the immature fruit of *S. grosvenorii*. Studies have shown that it inhibits the IL-9/IL-9 receptor pathway, Thus, the activities of trypsin and cathepsin B in pancreatic acinar cell line AR42J and primary acinar cells induced by nucleoprotein and LPS were attenuated (Xiao et al., 2020). Moreover, this modulation occurs in a dose- and time-dependent manner (Xiao et al., 2020). Therefore, Mog IIE is considered as a potential candidate for ameliorating pancreatitis.

*S. grosvenorii* polysaccharide SGP-1-1 can reduce the expression of IL-6 and TNF-α cytokines to treat the inflammation associated with diabetic nephropathy. This effect was mediated by down-regulating the mRNA and protein levels of Toll-like receptor 4 (TLR4) and its downstream protein kinase NF-κB p65 (Gong et al., 2021).

In summary, *S. grosvenorii* has a wide range of anti-inflammatory activities, and its extracts (SGEs), residue extracts (SGREs), MGEs, Mog V, 11-O-Mog V, Mog IIE, and polysaccharides have all demonstrated anti-inflammatory effects. Mog V, the most abundant compound in *S. grosvenorii*, has been especially effective in treating both respiratory and central system inflammation. The active compounds in *S. grosvenorii* have been shown to inhibit inflammation by interfering with the activation of the following pathways, including but not limited to the following: ① PI3K/Akt/IKK/NF-κB; ② MAPK/NF-κB pathway; ③ TLR4-MyD88 cascade; ④ AMPK/AKT-Nrf2 signaling axis; ⑤ JAK-STAT pathway. Moving forward, further research is needed to fully elucidate the specific mechanisms underlying *S. grosvenorii*’s anti-inflammatory properties.

### 3.4 Anti-aging and anti-oxidative stress

Oxidative stress is the excessive production of reactive oxygen species (ROS) such as superoxide anion free radicals, hydroxyl free radicals and hydrogen peroxide in the body when the body is subjected to various harmful stimuli, which breaks the balance between oxidation and antioxidant systems in the body, leading to tissue damage, aging and a variety of diseases (Golden et al., 2002; Kregel and Zhang, 2007). As an important antioxidant enzyme in the body, superoxide dismutase (SOD) can convert superoxide anion free radicals into hydrogen peroxide and oxygen ions, thereby reducing lipid peroxidation (McCord and Edeas, 2005). Malondialdehyde (MDA) is one of free radicals in the body cause lipid peroxidation product, its content can be used to judge the body indirectly the extent of lipid peroxidation damage and aging (Barrera et al., 2018).

D-galactose is a widely used reagent to study the antioxidant aging effect *in vivo*. *Drosophila* has similar aging genes to humans, so it is often used in experimental studies of aging life span. Some studies have shown that Mog can increase SOD activity and reduce MDA content in brain tissue and serum of aging mice induced by D-galactose (Xiao et al., 2014). In addition, it can significantly prolong *drosophila* life expectancy and the highest life (Xiao et al., 2014). This suggests that *S. grosvenorii* has a role in delaying aging. Another study explored the mechanism by which *S. grosvenorii* delayed aging. The results of free radical scavenging experiments and autooxidative hemolysis experiments of rat erythrocytes showed that SGEs had a strong ability to scavenge hydroxyl free radicals and superoxide anion free radicals, and could effectively inhibit the production of MDA in the process of autooxidative hemolysis of rat erythrocytes and liver lipid peroxidation in a dose-dependent manner (Qi et al., 2006). It is worth noting that the important antioxidant active ingredient in SGEs may be Mog V (Qi et al., 2006).

*In vitro* maturation (IVM) of oocytes is one of the most important steps in the *in vitro* production (IVP) of human and livestock embryos. Addition of Mog V to IVM medium has demonstrated a substantial augmentation of oocyte maturation, attributable to its facilitative role in first polar body extrusion by enhancing mitochondrial biosynthesis as well as reducing intracellular ROS accumulation (Nie et al., 2020). After 44 h of *in vitro* maturation, Mog V added to the first polar body of oocytes, sustained 24 h *in vitro,* has exhibited commendable proficiency in upholding conventional oocyte morphology and nascent embryonic developmental competence. Mog V also led to a reduction in ROS levels, spindle formation, and anomalous chromosomal disposition (Nie et al., 2019). What is noteworthy is that Mog V may improve oocyte quality by upregulating silencing message regulator 1 (SIRT1) to reduce oxidative stress during *in vitro* aging (Nie et al., 2019).

An innovative polysaccharide, denoted as SGP and characterized by a molecular weight of 1.93 × 10^3^ KDa, has been successfully extracted from residual components of *S. grosvenorii*. This compound has evinced robust *in vitro* antioxidant activity, particularly in terms of its proficiency in scavenging DPPH radicals. Moreover, SGP has demonstrated dose-dependent mitigation of ROS levels, apoptotic occurrences, and the proportion of necrotic cells in PC12 cells (a rat adrenal medullary pheochromocytoma cell line) (Zhu et al., 2020). In addition, SGP-1-1 stimulates the production of SOD and reduces the production of MDA by down-regulating the activation of TLR4/NF-κB p65 pathway to reduce oxidative stress caused by diabetic nephropathy (Gong et al., 2021).

Moving on to the realm of edibles, MGEs has emerged as a promising natural antioxidant for the preservation of quality in meat and meat products. The study confirmed the role of MGEs as inherently natural antioxidant in the preparation of dried mince. The natural antioxidants in MGEs not only have a protective effect against lipid and protein oxidation under vacuum storage conditions, but also maintain the reddish-brown character of dried minced meat (Cheng et al., 2017).

Taken together, existing studies indicate that the active substances in *S. grosvenorii* responsible for its anti-aging effects are Mog V and polysaccharides. The anti-aging mechanism of *S. grosvenorii* may be related to maintaining the balance of free radical metabolism by improving the activity of antioxidant enzymes in the body, removing excessive free radicals in the body, and reducing the lipid peroxidation of biofilm caused by free radicals, thereby protecting the normal metabolism of the body. In the future, there is a need to further verify whether other active components of *S. grosvenorii* possess anti-aging properties.

### 3.5 Reduce blood sugar

Diabetes mellitus, a pervasive chronic ailment, manifests as an endocrine and metabolic disorder characterized by hyperglycemia, late microvascular and macrovascular complications, of which diabetic nephropathy emerges as a profoundly detrimental consequence (Kaul et al., 2013). SGEs, MGEs, and multiple Mogs may inhibit diabetes-induced hyperglycemia and contribute to the prevention of diabetic complications associated with oxidative stress and hyperlipidemia.

The potential of SGEs to manifest anti-diabetic traits on spontaneously diabetic Goto-Kakizaki rats has been underscored by augmented fasting state insulin secretion, enhanced renal functionality, and fortified hepatic and plasma antioxidant attributes (Suzuki et al., 2007).

An experiment conducted on mice with alloxan-induced diabetic revealed that administering MGEs orally at different doses (100, 300, and 500 mg/kg) over a 4-week period led to significant reductions in blood glucose levels. Additionally, this treatment resulted in decreased levels of serum total cholesterol (TC), triglycerides (TG), and hepatic MDA, while increasing serum high-density lipoprotein cholesterol (HDL-C) levels and restoring hepatic antioxidant enzyme activities. Among these doses, the group receiving MGEs at 100 mg/kg exhibited the most pronounced hypoglycemic, hypolipidemic, and antioxidant effects, akin to the impact of Xiao Ke Wan-pills (at a dose of 894 mg/kg) (Qi et al., 2008). Another study demonstrated that diabetic mice given 150 or 300 mg/kg of MGEs for 5 consecutive weeks experienced a dose-dependent reduction in fasting blood glucose (FBG), glycated serum protein (GSP), serum insulin, homeostasis model assessment of insulin resistance (HOMA-IR), and serum atherogenic lipid profiles without any notable changes in their body weights or energy intake (Liu et al., 2019).

Mogs can prevent the development of diabetic nephropathy through antioxidant effects associated with inhibition the activation of heme oxygenase-1 (HO-1) and has no toxic effects in normal mice (Song et al., 2007). Moreover, *S. grosvenorii* crude extract (SG-ex) and its main sweet-tasting ingredient, monosodium glutamate (MSG), Mog V, as well as some minor ingredients such as Mog IV, siamenoside I, and Mog III exert anti-hyperglycemic effects in rats through the inhibition of wheat glycoenzyme activity (Suzuki et al., 2005). Professor Li Xiaobo’s team is very accomplished in the research on the anti-blood sugar of *S. grosvenorii*. They have developed a highly sensitive and rapid ultra-performance liquid chromatography/quadrupole time-of-flight mass spectrometry (UPLC-Q-TOF/MS) method, n conjunction with the Metabolynx™ software and mass defect filtering, to detect and characterize metabolites in the plasma, urine, bile, and feces of healthy rats and rats with type 2 diabetes mellitus (T2DM). Their study found that the main metabolic transformations of Mog V were achieved through dehydrogenation, deoxidation, oxidation, and isomerization (Zhou et al., 2018a). Additionally, they isolated low-polar *Siraitia grosvenorii* glycosides (L-SGgly), containing 1-3 glucose residues, from the 50% ethanol extract. L-SGgly contained 11-oxo-mog V, Mog V, Mog III, Mog IIE, Mog IIIA1, Mog IIA1, and Mog IA1, with the total content of Mogs being 54.4%, including 15.7% Mog IIA1 and 12.6% Mog IA1 (Zhang et al., 2020a). Compared to other parts of the LHG extract, L-SGgly exhibited promising effects on obese T2DM rats, resulting in a significant reduction in FBG levels and an improvement in insulin resistance. The anti-diabetic activity of L-SGgly may be attributed to increased glucagon-like peptide-1 (GLP-1) levels and activation of hepatic AMPK in T2DM rats (Zhang et al., 2020a). Furthermore, Li Xiaobo’s study suggests that the gut microbiota and their metabolites, such as Elusimicrobium, Lachnospiraceae_UCG-004, acetate, butyrate, and 1β-hydroxycholic acid, are potential dominant bacteria and biomarkers for SGEs and L-SGgly in reducing blood glucose and insulin resistance in T2DM rats (Zhang et al., 2021).

To sum up, *S. grosvenorii* is a promising dietary supplement for blood sugar control, especially for diabetics with a sweet tooth. Because it can both address diabetics’ desire for sweetness and help lower blood sugar. The hypoglycemic mechanism of *S. grosvenorii* appears to be linked to its ability to combat oxidative stress, inhibit the activity of wheat glycoenzyme, and activate AMPK in the liver. However, the specific mechanisms responsible for the hypoglycemic effects of *S. grosvenorii* are not yet fully understood and require further investigation.

### 3.6 Inhibit lipid deposition and obesity

*S. grosvenorii* exhibits promising potential in mitigating non-alcoholic fatty liver disease. Activation of the 5′ AMP-activated protein kinase (AMPK) signaling pathway within the hepatic milieu of diabetic murine models displayed a discernible MGEs dose-dependent pattern. Persistent administration of a MGEs at a concentration of 300 mg/kg over a span of 5 weeks yielded noteworthy amelioration. This was evident in the amelioration of hepatocyte polymorphism, mitigation of lipid accrual, and attenuation of steatosis, with restorative trends approximating basal states, as elucidated in the scholarly discourse (Liu et al., 2019). Mog V can improve the imbalance between lipid acquisition (*de novo* synthesis of lipids) and lipid clearance (lipolysis and fatty acid oxidation) by activating AMPK and subsequently exerting downstream regulation of SREBP1, PPAR-c and PPAR-α, thereby improving the liver steatosis induced by high fat diet in mice (Liu et al., 2019). Furthermore, the introduction of Nanoselenium *S. grosvenorii* (NSG) demonstrated multifaceted effects. NSG can not only enhance the diversity of intestinal flora and change its composition, but also significantly reduce the degradation of liver lysine metabolism and inhibit fatty acid β-oxidation, thus having a positive effect on obesity (Wang et al., 2023).

In summation, these discerning findings underscore the potential of *S. grosvenorii* and its derivative NSG as therapeutic agents to counteract non-alcoholic fatty liver disease and obesity-related pathways. Further exploration is needed to determine whether *S. grosvenorii* regulates pathways beyond AMPK and to unveil more specific regulatory mechanisms.

### 3.7 Anti-depression-like effect

Recent investigations have unveiled the involvement of *S. grosvenorii* in the modulation of neurological disorders. Mog V has demonstrated a safeguarding capacity against corticosterone-induced damage in PC12 cells (Liu et al., 2023a). Furthermore, administration of Mog V has exhibited the potential to mitigate depressive manifestations within the chronic unpredictable mild stress (CUMS) depression model mice. Mechanistically, this intervention exerts an antidepressant influence by suppressing pathways related to inflammation and oxidative stress, along with the brain-derived neurotrophic factor (BDNF)/tyrosine kinase receptor B (TrkB)/AKT pathway (Liu et al., 2023a). These compelling revelations introduce innovative perspectives for the identification of emerging strategies in the realm of antidepressant interventions.

### 3.8 Anti-fatigue effects

Single-session exhaustive exercise as well as repeated exhaustive exercise have been shown to elicit a decrease in the enzymatic activities of SOD, catalase (CAT), and glutathione peroxidase (GSH-PX). Simultaneously, an elevation in MDA and calcium ion (Ca^2+^) levels has been observed. This intricate interplay suggests that the potential anti-fatigue mechanism exhibited by *S. grosvenorii* and its extracts could be attributed to the suppression of oxidative stress and lipid peroxidation pathways.

SGEs have been found to dose-dependently enhance the physiological functions of Kunming mice during incremental load training. These enhancements encompass aspects such as exercise capacity, hypoxia and heat tolerance, and the attenuation of exercise-induced fatigue. The optimal dose was 15.0g/k·w·d. Notably, surpassing this dose threshold led to a diminishing impact on the mice’s physiological improvements (Yao et al., 2007). One important observation has been made regarding the administration of SGEs by gavage at different doses (100 mg/kg, 200 mg/kg, and 400 mg/kg) for 28 consecutive days. Evidently, such intervention imparts an extension to the swimming capacity of murine subjects. Concurrently, augmented hepatic and muscular glycogen reservoirs are discerned, accompanied by a concomitant attenuation of both blood lactic acid and serum urea nitrogen concentrations. Notably, the increased endurance of physical fatigue in mice showed a clear SGEs dose-dependent effect (Liu et al., 2013a).

Mogrol, an end product of Mog metabolism, has been shown to exert anti-fatigue effects by augmenting the relative protein expression of phosphorylated AMP-activated protein kinase alpha (p-AMPKα), AMPKα, peroxisome proliferator-activated receptor gamma coactivator 1-alpha (PGC-1α), and mitochondrial transcription factor A (TFAM) within the skeletal muscle of fatigued rats (Guo and Wen, 2022). Flavonoids have been documented to enhance the exercise capacity of male SD rats by fostering augmented blood perfusion and oxygen transport capabilities within the muscle tissue (Chen et al., 2020).

Leaf-derived flavonoids display a multifaceted anti-fatigue effect, encompassing the elevation of antioxidant enzyme activities, effective scavenging of excessive free radicals arising from exhaustive exercise, curbing lipid peroxidation, safeguarding myocardial mitochondria (Xie, 2018), and restraining apoptotic processes (Guo and Peng, 2012).

Mogs exhibit a pronounced capacity to bolster anti-fatigue potential and hypoxia tolerance in mice. These benefits can be attributed to heightened glycogen storage and accelerated lactate metabolism, pivotal factors contributing to improved endurance (Xia et al., 2012).

In summation, these studies demonstrate the potential utility of SGEs, mogrol, leaf-derived flavone, and Mogs, in combating fatigue and enhancing endurance in *S. grosvenorii*. The anti-fatigue effects demonstrated by *S. grosvenorii* and its derivatives manifest through a spectrum of mechanisms, including modulation of oxidative stress, enhancement of mitochondrial resilience, augmentation of vascular supply, and orchestrated protein expression. However, further studies are needed to reveal the specific molecules targeted by these active ingredients.

### 3.9 Anti-schizophrenia

Schizophrenia is a kind of the signs and symptoms of unknown origin, main characteristic is showed signs of mental illness (Insel, 2010). MK801 to glutamate-N-methyl-D-aspartate (NMDA) receptor non-competitive antagonist, is currently the animal models of common tools used to construct the schizophrenia (Insel, 2010). Prevent prepulse inhibition (PPI) reflects the preattentional regulation of midbrain information filtering by the forebrain neural circuit. Decreased PPI means that the individual’s filtering mechanism of external environment information is decreased, which leads to information input overload and damage to the attention allocation process (Insel, 2010). Therefore, reducing PPI deficiency can be used as one of the indicators for improving psychiatric symptoms in the treatment of schizophrenia.

*S. grosvenorii* demonstrates a capacity for the modulation of schizophrenia. Noteworthy in this context is the efficacy of Mog V, has a alleviating effect on PPI deficits and social withdrawal induced by MK801(Ju et al., 2015; Ju et al., 2020). In addition, Mog V treatment reduced the total distance of movement and the number of spontaneous movements in MK801-induced schizophrenic mice (Ju et al., 2015). Moreover, Mog V has demonstrated a propensity to enhance cellular and neurochemical responses to MK801 within the confines of the medial prefrontal cortex (mPFC), as evidenced by empirical observations (Ju et al., 2020). Mog V, in concert with its metabolite 11-oxo-mogrol, serves as a preventive measure against MK-801-driven neuronal injury, largely attributed to its facilitation of neurite outgrowth and concurrent inhibition of cellular apoptosis, alongside the suppression of [Ca^2+^]i release (Ju et al., 2020). In addition, 11-oxo-mogrol regulates schizophrenia by reversing MK801-induced inactivation of AKT and phosphorylation of mammalian target of rapamycin (mTOR) (Ju et al., 2020).

### 3.10 Anti-Parkinson’s disease (PD)

PD is the second most common neurodegenerative disease in the world, which is common in middle-aged and elderly people. Its clinical manifestations are mainly bradykinesia, muscle stiffness and resting tremor, as well as non-motor symptoms such as anxiety, depression and cognitive impairment. Its main pathological feature is the degenerative loss of dopaminergic neurons in the substantia nigra region of the brain (Kalia and Lang, 2015).

Both Mog V and mogrol could significantly protect SY5Y cells from MPP^+^ -induced injury *in vitro* (Tang, 2021). *In vivo* experiments showed that Mog V could effectively improve the motor deficits and pathological changes of the brain in MPTP-induced PD mice, and had a significant protective effect on neurons in the substantia nigra region of mice. To the little mouse nigra district organization, according to the results of test of metabonomics in significant differences in metabolic substances after the treatment has 2 - D - hydrogen sphingosine, n-acetyl - L - glutamic acid, trimethylamine oxide, hexadecanoic acid, 9 - oleic aldehydes and 1, 11-11 of carbon dibasic acid. The recovered metabolic pathways were mainly focused on lipid and sphingolipid metabolism and amino acid synthesis. This suggests that Mog V plays a neuroprotective role mainly by regulating amino acid synthesis, lipid metabolism and gut-brain axis homeostasis, thereby alleviating mitochondrial dysfunction and substantia nigra neuronal loss (Tang, 2021). A recent investigation has exhibited that the administration of a 10 mg/kg dose of Mog V yielded a marked reversal of motor deficits and the consequential dopaminergic neuronal impairment triggered by rotenone in mice with PD (Luo et al., 2022). The mechanism may be that Mog V reduces overproduction of ROS in a dose-dependent manner, restores mitochondrial membrane potential, increases oxygen consumption rate and adenosine triphosphate (ATP) production, leading to a decrease in the number of apoptotic cells (Luo et al., 2022). Moreover, it is discerned that Mog V exhibits a safeguarding influence against PD, demonstrable in both the substantia nigra of mouse and SH-SY5Y cell line, by orchestrating a downregulation of SIRT3 acetylation (Luo et al., 2022). Furthermore, Mog V appears to intercede the injury-induced activity of the pivotal mitochondrial antioxidant enzyme, SOD2, thereby fortifying its neuroprotective impact (Luo et al., 2022).

### 3.11 Anti-fibrosis

Fibrosis-related diseases such as NASH, idiopathic pulmonary fibrosis (IPF), and myocardial fibrosis (MF) are all characterized by excessive deposition of extracellular matrix (ECM) (Jellis et al., 2010; Lederer and Martinez, 2018; Sheka et al., 2020). Fibrosis is the final pathological process of many diseases, however, only a limited number of drugs have shown effectiveness in reversing this condition.

The role of SGEs in the treatment of liver fibrosis has been proven. A study established a rat nutritional model of NASH by long-term feeding of CDAA to SD rats, and overcame dietary antagonism by feeding mice a high-fat diet lacking choline, low methionine, L-amino acid restriction, and free of trans fatty acids (CDAA-HF-T (-)). The majority of the phenotypic and mechanistic traits of human NASH are replicated in these models, including the rapid induction of fibrosis and the proliferation of lesions in the liver. SGEs prevent NASH in CDAA-HF-T (-) mice by inhibiting fibrosis without changing steatosis (Suzuki-Kemuriyama et al., 2021). It is possible that SGEs prevent liver fibrosis by preventing the NF-κB pathway from being active (Uno et al., 2023).

Within *S. grosvenorii*, active compounds with anti-fibrotic properties include the bitter components Mog IIIE and morgol. Mog IIIE has been found to reduce lung inflammation and inhibit ECM deposition by modulating the TLR4/MyD88-MAPK signaling cascade. In fact, at a dosage of 20 mg/kg, Mog IIIE was found to be more effective than Prednisone at 6.5 mg/kg in inhibiting pulmonary fibrosis (Tao et al., 2017). Additionally, research has demonstrated that Mog IIIE can reduce liver fibrosis by inhibiting the TLR4/MyD88/NF-κB signaling pathway (Yanan et al., 2023). Mogrol isolated from *S*. *grosvenorii* can not only reduce lung inflammation by activating AMPK, but also inhibit TGF-β1-induced mesenchymal transformation of alveolar epithelial cells and transdifferentiation of myofibroblasts through activation of the TGF-β1/Smad2/3 signaling pathway, thus exerting antifibrotic effects (Liu et al., 2021a). Notably, a dosage of 10 mg/kg of mogrol demonstrated better inhibition of bleomycin-induced pulmonary fibrosis compared to 40 mg/kg of nintedanib (Liu et al., 2021a).

Interestingly, it remains unclear from existing literature whether Mog V, which has the highest content among these components, possesses anti-fibrotic potential. It is worth noting that *S. grosvenorii*, may potentially contribute to the treatment, alleviation, or even reversal of fibrosis in the future. Before this can be done, further research and studies are needed to explore its potential therapeutic benefits in this regard.

### 3.12 Anti-cancer

Prostate cancer and bladder cancer stand as the prevailing malignancies within the domain of urology. Despite the array of available therapeutic interventions for these conditions, their efficacy remains suboptimal. The therapeutic effects of SGEs and multiple Mogs on different cancers have been revealed several times.

Mechanistically, pretreatment of murine skin with SGEs exhibited attenuation of TPA-induced activation of key signaling molecules, including extracellular signal-regulated kinase (ERK)1/2, p38 MAPK, c-Jun N-terminal kinase (JNK)1/2, PI3K, and Akt. This orchestrated reduction curtailed TPA-induced nuclear translocation of NF-κB subunits and phosphorylation of IkBa and p65, thereby culminating in a substantial impediment of 7,12-DMBA-TPA-evoked cutaneous tumor genesis (Weerawatanakorn et al., 2014).

LLE and Mog (LLE is a liquid form with Mog; and Mog is a highly purified Mog powder) can significantly reduce the viability of PC-3 (human prostate cancer cells) and T24 cells (human bladder transitional cell cancer cells), elicited a pronounced induction of G1 phase cell cycle arrest, induce endoplasmic reticulum stress and apoptosis, and thus treat prostate cancer and bladder cancer (Haung et al., 2023). Mog V and 11-oxo-Mog V showed a significant inhibitory effect on the two-stage carcinogenesis test of mouse skin tumors induced by peroxynitrite (ONOO2) as an initiator and TPA as a promoter (Takasaki et al., 2003). Moreover, 11-oxo-Mog V showcased substantial inhibition against two-stage skin carcinogenesis induced by 7,12-dimethylbenz [α]-anthracene (DMBA) and TPA (Takasaki et al., 2003), further accentuating its potential.

Furthermore, there is a investigation unveiled that within *S. grosvenorii*, 18 triterpenoids and 11-oxo-mogrol exhibited the capacity to curtail the generation of Epstein-Barr virus early antigen (EBV-EA) within Raji cells, incited by TPA (Ukiya et al., 2002). Researchers have tested whether mogrol has an effect on the proliferation of human prostate cancer cell line DU145, human liver cancer cell line HepG2, human lung cancer cell line A549, human nasopharyngeal carcinoma cell lines CNE1 and CNE2. The results showed that mogrol had obvious inhibitory effect on the proliferation of the above cells, but the inhibitory effect on the proliferation of human nasopharyngeal carcinoma CNE1 cells was the strongest. Mogrol has been shown to exert anti-tumor effects by inducing apoptosis (Fu et al., 2016) and G0/G1 cell cycle arrest (Liu et al., 2015), thereby inhibiting the proliferation of tumor cells.

The aforementioned studies have demonstrated that Mog V, 11-oxo-mog V, triterpenoids, 11-oxo-mogrol, and mogrol derived from *S. grosvenorii* exhibit anti-tumor effects. These effects are likely mediated by reducing cell viability, inducing G1-phase cell-cycle arrest, and triggering endoplasmic reticulum stress and apoptosis.

Although *S. grosvenorii* is traditionally known for its expectorant and cough suppressant properties, the above studies all suggest that *S. grosvenorii* has much more pharmacological effects than that. Among its various constituents, Mog V has been extensively studied, yet it remains uncertain whether any particular pharmacological effect outweighs the others. Furthermore, trace components like Mog IIIE and SGP-1-1 have also exhibited promising anti-fibrotic and anti-inflammatory effects. Despite the discovery of various pharmacological effects of *S. grosvenorii* and its promising efficacy in treating different diseases, it is important to note that these findings are primarily based on animal and cellular experiments. Therefore, it is crucial to conduct sufficient clinical studies to validate the therapeutic effects of *S. grosvenorii*. Furthermore, the majority of experiments conducted utilized either total Mogs or SGEs. However, there remains a lack of clarity regarding which specific monomeric substance is responsible for exerting distinct pharmacological effects. It is essential to determine which monomer exhibits the highest pharmacological activity and whether any synergistic or antagonistic effects exist among these monomers. Additionally, it is also necessary to compare the therapeutic effect of a single monomer of *S. grosvenorii* with a positive control. Moreover, it is crucial to gain a comprehensive understanding of the precise mechanisms and target molecules involved in regulating diseases. To address these critical questions, further experimental data will be necessary in future studies.

## 4 Toxicology

Although *S*. *grosvenorii* has been consumed for more than 300 years, elucidation of its toxicity is necessary before its large-scale use in food and medicine.

At present, some research teams have used acute toxicity test, long toxicity test, bone marrow micronucleus test, sperm abnormality test and reproductive and measuring lymphatic organ index to scrutinized the toxicological ramifications of *S. grosvenorii* and its extracts. Some researchers administered 3 g/mL of aqueous extract of *S. grosvenorii* to Kunming mice by gavage at a dose of 120 g/kg of dried fruit. After administration, although the mice’s intake of water and food was significantly reduced, this reduction was transient and did not cause lasting health effects. In the following 2 weeks, the mice maintained normal behavioral tendencies, sensory responses, fur quality, and intake of nutrients and water. Furthermore, the mice showed no obvious signs of toxicity, did not die, and the structural integrity of major organs was not compromised (Zhang et al., 2011). Bone marrow micronucleus frequencies, sperm abnormality coefficients, and reproductive and lymphoid organ indices were also virtually identical to those of untreated mice (Zhang et al., 2011).

Despite these experimental evidence, also cannot easily think of *S. grosvenorii* no pathogenic potential harm to the human body. Noteworthy, is the observation from a distinct study elucidating that while acute toxicity evaluations of Mogs (total glycosides ≥80%) in male Kunming mice proved non-deleterious, escalated doses (≥5 g/kg) elicited discernible, albeit modest, effects on micronucleus frequency and sperm aberrations (Liu et al., 2013b). In addition, some research teams did a screening experiment on the antitussive active ingredients of *S. grosvenorii* and found that when the dose of *S. grosvenorii* water decoction reached 50 g/kg of crude drug, some animals were in a poorer condition than control group mice (Liu et al., 2007), so the possibility of adverse stimulation of larger doses of *S. grosvenorii* could not be excluded.

Hence, whether harnessed for therapeutic interventions or harnessed as an innate nutritive sweetening agent, the possible genotoxic propensity of *S. grosvenorii* at high doses should be considered with caution.

## 5 The current status of exploitation and utilization, application and development challenges of *S. grosvenorii* resources

### 5.1 Exploitation and utilization status

*S. grosvenorii* is exclusively grown in China (He et al., 2012). With special requirements on ecological conditions, its distribution is limited to certain regions in provinces such as Guangxi, Guangdong, Hunan, Jiangxi, with Guangxi accounting for approximately 80% of the cultivated area in China (Li and Lan, 2019). Currently, the primary focus of cultivation for *S. grosvenorii* revolves around the green fruit variety, followed by La Jiang fruit and long beach fruit (Tang, 2023b). The planting of *S. grosvenorii* typically occurs through the use of cutting seedings, which takes place annually from mid-March to mid-April (Tang, 2023b). Prior to planting, it is necessary to construct a shed (Tang, 2023b). Once the *S. grosvenorii* seedlings reach a height of 15 cm, the main vine is carefully chosen and transferred into the shed for pruning (Tang, 2023b). Artificial pollination becomes imperative after flowering (Tang, 2023b). Traditional cultivation of *S. grosvenorii* faced challenges including low yield, low seedling survival rate, and susceptibility to pests and diseases. However, advancements in technology have successfully addressed these issues, resulting in a significant increase in the yield of *S. grosvenorii* seedlings from less than 20% to over 90% (Li and Lan, 2019). The new varieties of *S. grosvenorii* histocultured seedlings have many excellent characteristics such as no virus, good disease resistance, and strong adaptability. The conquest of this technical problem has greatly reduced the cost of labor and agricultural materials. Overcoming this technical obstacle has not only reduced labor and agricultural costs but has also expanded the cultivation areas beyond mountainous regions (Li and Lan, 2019). Guilin City, known for being the largest cultivation area for *S. grosvenorii*, recorded a cultivation area of 225,500 mu and an LHG output of 2,252 million in 2022 (Qin et al., 2023).

### 5.2 Application

Traditionally, *S. grosvenorii* is used as a traditional Chinese medicine for the treatment of lung fire and dry cough, sore throat and loss of voice, and intestinal dryness and constipation (Jiang et al., 2015). Currently, its primary application lies in its use as a natural sweetener. In 1995, the United States Food and Drug Administration (FDA) approved the application of Mog in food products, and after obtaining the United States FDA Generally Recognized as Safe (GRAS) certification in 2011, *S. grosvenorii* extracts/processed products have successfully entered United States market (Pawar et al., 2013). In China, Mog was approved as a food additive at the 17th meeting of the National Food Additives Committee in 1996, and can be used as a sweetener to partially or completely replace sucrose (Chen et al., 2022), The official implementation of the “Food Additives of Mog” in 2017 further clarified its application in Chinese market (Chen et al., 2022). Several other countries, including Japan, South Korea, and the United Kingdom, also permit the use of Mog as a food additive (Chen et al., 2022).

Nowadays, Mog substances are widely used in the food industry. From 2016 to 2020, the amount of Mogs utilized in the United States market has steadily increased, with the global Mog market expected to exceed USD 1 billion, growing at a compound annual growth rate of over 40% from its approximate value of USD 250 million in 2016 (Chen et al., 2022). In the food industry, LHG is employed to produce a variety of consumables, including LHG-fermented wine, LHG cake (Hong et al., 2021; Liu et al., 2023b), LHG preserved fruit, and LHG fruit compound beverages (Li et al., 2006a).

*S. grosvenorii* has a certain presence in the healthcare industries. In the pharmaceutical field, products like *Luo Han Guo Paoteng Tablet* and *Luo Han Guo yanhou Tablet* have been developed to provide relief from pharyngeal discomfort (Jin et al., 1997; Jiang et al., 2011; Chen et al., 2015). *Luo Han Guo Lvcha Granules* are used to boost immunity, *Luo Han Guo Sydney Cream* and *Jinyinhua Luo Han Guo Lozenges* are used to treat sore throats (Zhang et al., 2017). Additionally, when combined with ingredients like hawthorn, yam, and tangerini peel, LHG can be used to create a health drink that strengthens the stomach and aids digestion (Sun, 2022). In China and Japan, Mog extract has been approved as a healthy alternative to sugar for obese and diabetic patients (Gong et al., 2019).

In recent years, the livestock, tobacco and cosmetics industries also witnesses *S. grosvenorii*’s influence. As its residual components find utility in enhancing feed digestibility, augmenting poultry growth, and ameliorating slaughter performance, evident in the supplementation of Guangxi hemp chicken diets with *S. grosvenorii* residues (Xu et al., 2022a; Wang et al., 2023; Xiao et al., 2023). Furthermore, incorporating *S. grosvenorii* flavoring into smoking products not only enhances the sensory experience but also helps protect the health of smokers by reducing harmful pulmonary consequences, alleviating throat irritation, and even aiding smoking cessation (Li et al., 2006a; Zhang et al., 2018; Wu et al., 2022b). Moreover, there are many patented products of *S. grosvenorii* that find applications in the cosmetic industry due to their skin whitening and moisturizing effects (Zhang and Zhang, 2011; Yang, 2022).

Although the application scope of *S. grosvenorii* covers a wide range of food, drugs, cosmetics, health products, animal feed and other fields have broad prospects. A problem that cannot be ignored is that *S. grosvenorii* extracts/processed products are still cold in the Chinese market where they originate. There are many challenges that need to be solved in order to further popularize it in the market.

### 5.3 Development challenges

The development and widespread use of *S. grosvenorii* as a sweetener and functional product in the market is facing five urgent challenges.

Firstly, the supply of raw materials of *S. grosvenorii* is insufficient, and the production level of *S. grosvenorii* extract products is not high. *S. grosvenorii* extract products are made from the natural *S. grosvenorii* plant, which has distinct cyclical, regional, and seasonal characteristics in terms of planting, harvesting, and acquisition. The availability of raw materials is influenced by various factors such as geographical limitations and climate changes, making it vulnerable to price fluctuations and an unstable supply (Zhao, 2023). *S. grosvenorii* is a crop that requires ample sunlight during its growth process, but prolonged exposure to intense light and low temperatures can hinder its growth. As a result, the cultivation area for *S. grosvenorii is limited* (Tang, 2023a). Currently, most *S. grosvenorii* cultivation is carried out by individual farmers without proper scientific planning (Tang, 2023a), leading to issues like blindly expanding cultivation trends and inconsistent production standards. Additionally, the lack of effective supervision in *S. grosvenorii* cultivation has prevented standardized operations, deviating from the intended goals. These problems directly hinder the transformation of the *S. grosvenorii* industry’s advantages into economic benefits.

Secondly, intense competition exists among sweetener products. The natural sweeteners market is currently flooded with a wide range of competing products, and unfortunately, Mog faces certain drawbacks regarding its price, stability, and taste when compared to other popular high-frequency sweeteners. The extraction cost of Mog is relatively high, leading to end-product prices that are higher than similar alternatives (Zhao, 2023). In addition, the traditional processing methods used in the original production areas of *S. grosvenorii*, such as firewood baking, have resulted in a distinct roasted taste that is not easily recognized by consumers. Moreover, the purity of available Mogs in the market is generally low, ranging from 25% to 50%, and this low purity level is associated with an unpleasant taste (Huang et al., 2021). To mask its distinctive odor, Mog is often combined with other sweeteners like erythritol, further increasing costs. As a result, in recent years, major food and beverage companies have shown a preference for Steviol glycosides, another natural high-frequency sweetener, instead of *S. grosvenorii* (Zhao, 2023).

Thirdly, there is limited diversity in the range of *S. grosvenorii* end products. Mog is primarily used as a sweetener, resulting in its predominant existence as an additive in the market. Moreover, the history of using Mog as a sweetener is relatively short, and research on its pharmacological function and toxicology is still in its early stages. Therefore, the number and variety of end products containing *S. grosvenorii* as the main ingredient are still insufficient. Between 2014 and 2018, although SGEs were used in approximately 2,507 food, beverage, dairy, and other products worldwide, the number of food and beverage products containing SGEs in 2018 was actually lower than in 2014 (Li and Lan, 2019). Due to the high cost of SGE and its lack of competitiveness in terms of price, it faces challenges in achieving rapid growth both domestically and internationally.

Fourthly, the market recognition of *S. grosvenorii* products is relatively low. As a specialty cash crop, consumers are not yet familiar with the efficacy of *S. grosvenorii*, and the lack of branding and market education has resulted in a limited understanding and trust in its characteristics, functions, and advantages. This lack of awareness makes it difficult to generate consumer demand and preference for *S. grosvenorii* products. Despite the abundance of literature supporting the pharmacological efficacy of *S. grosvenorii*, insufficient publicity and popularization efforts have created the impression among consumers that there is no difference between Mog as a sweetener and other natural sweeteners.

Fifthly, the marketing channels for *S. grosvenorii* products are relatively narrow. Most *S. grosvenorii* products are currently marketed through direct sales channels, resulting in high operating and management expenses and significant labor costs. This has led to increased prices for the extract products, which were already at a disadvantage when compared to similar alternatives (Zhao, 2023). Furthermore, the lack of promotional efforts by distributors has severely restricted the speed of *S. grosvenorii*’s end products’ rollout. Although in recent years, live broadcasting has emerged as a new sales method, some sellers have tried to promote *S. grosvenorii* products through this platform. However, due to the low market awareness of SGEs products, this sales method has not proven as effective as expected, despite its convenience.

## 6 Strategies and methods of intensive development and utilization of *S. grosvenorii*

In order to further expand both domestic and overseas markets for *S. grosvenorii* as a sweetener or functional product, it is crucial to implement effective strategies and methods for its intensive development and utilization.

### 6.1 Increase the research and development of new *S. grosvenorii* varieties to improve the quality of *S. grosvenorii*

While the existing varieties of *S. grosvenorii* have addressed issues such as low survival rates, pests, diseases, and low yield, there are still certain challenges that distinguish it from *Stevia*, another natural sweetener plant. These include a lower content of effective components, germplasm degeneration, mixing, and notable problems related to continuous cropping. These factors contribute to the degradation of the soil’s ecological environment. Therefore, it is crucial to continue in-depth research to develop new *S. grosvenorii* varieties that offer high yield and quality, strong resistance, adaptability, and reduced planting and management costs. This will involve optimizing the regional and seasonal layouts of *S. grosvenorii* varieties to enhance both yield and quality, as well as extending the availability of fresh fruits in the market.

### 6.2 Innovative planting mode and its supporting technology to reduce the cost of raw material planting

Moving forward, it is essential to standardize the planting mode for *S. grosvenorii*, while also accurately estimating market demand and avoiding blind production. Improving the intensity of planting will help to prevent the waste of raw materials and reduce planting costs. To achieve this, it is crucial to propose unified production and technical standards.

### 6.3 Optimize and improve the production process of *S. grosvenorii* deep-processed products

Looking ahead, it is crucial to focus on the development and application of *S. grosvenorii* as a natural sweetener in response to the growing global demand for sugar reduction and increased health consciousness. This can be achieved through extensive research on the development and utilization of the *S. grosvenorii* serie of natural sweetener products, with a particular emphasis on higher purity MGE. Expanding the application scope of Mog products and establishing a comprehensive support system will be key. Furthermore, there should be efforts to improve the processing techniques of existing *S. grosvenorii* products, aiming to reduce processing costs and develop efficient and labor-saving technological equipment. Streamlining the production process to achieve continuous and automated production will be crucial, ultimately optimizing human labor in the process.

### 6.4 Innovative research and development of new *S. grosvenorii* products to meet the differentiated needs of the market

As a dual-use traditional Chinese medicine, the application of medicines/healthcare products related to the pharmacological activities of *S. grosvenorii*, such as anti-inflammatory, antioxidant, anti-tumor, anti-fatigue, etc., has yet to be further researched. The pace of research should be accelerated to comprehensively develop the value of medicinal constituents of *S. grosvenorii*, and to obtain new products with the special efficacy of *S. grosvenorii*, so as to satisfy the differentiated needs of the market.

### 6.5 Increase the popularization and publicity of the efficacy of *S. grosvenorii*

In the future, it is vital to increase the cultural promotion of *S. grosvenorii* in order to enhance consumer awareness and understanding. Various strategies can be employed to achieve this, such as organizing *S. grosvenorii* cultural festivals, exhibitions, and establishing offline counters. Leveraging both traditional paper media and modern digital channels, public relations and publicity initiatives can be implemented. The focus of these efforts should center around highlighting the medicinal value of *S. grosvenorii* and its natural, healthy advantages as a sweetener. By emphasizing these key points to deepen consumer impressions of *S. grosvenorii* and its extracted products, ensuring that they are well-informed and more receptive to its benefits.

### 6.6 Optimizing prices and expanding distribution channels for *S. grosvenorii* products

In general, price plays a pivotal role in consumers’ decision-making process when it comes to purchasing a product. As a result, it becomes crucial to align the pricing of *S. grosvenorii* products with the prevailing market environment. This necessitates a thorough evaluation and adjustment of prices to ensure competitiveness. Moreover, it is imperative to establish a robust marketing system for physical stores to enhance the efficiency of product rollout. Simultaneously, optimizing online sales channels is equally important. By leveraging e-commerce platforms, social media, and various online networks, to effectively reach new customer segments, thereby capturing untapped market opportunities.

## 7 Conclusion

Based on our comprehensive understanding of *S. grosvenorii*, we now present a forward-looking analysis of the future direction of *S. grosvenorii* production in 2030. To ensure the sustainability of plant resources, the planting scale and area for *S. grosvenorii* will be expanded. Furthermore, the processing techniques will be optimized, yield and quality will be improved, freshness duration of LHG will be extended, and stability of the *S. grosvenorii* supply chain will be enhanced. In addition, the industry standards and norms will be established, a highly regarded and popular *S. grosvenorii* brand will emergence. The industrial production of high purity Mogs and the masking of the unique smell of Mogs will be realized. These developments will expand the market for *S. grosvenorii* as a sweetener. Since 2010, there has been a steady increase in the number of publications and citations on *S. grosvenorii*, particularly focusing on its health effects and biosynthesis of Mogs (Yeung, 2023). However, at present, there is limited clinical trial evidence regarding the pharmacological effects of *S. grosvenorii*. Therefore, it is reasonable to expect that by 2030, there will be a substantial amount of clinical data evaluating the overall pharmacological effects and toxicity of *S. grosvenorii*. This will shed light on the biological effects of the main chemical components within total Mogs of *S. grosvenorii*, as well as their interactions with other compounds.

Based on the existing research work, this review comprehensively summarizes the chemical composition, pharmacological effects, toxicology, status of resources development, and applications of *S. grosvenorii*. Additionally, this review offers strategies and methods of intensive development and utilization of *S. grosvenorii.* It is hoped that this paper will inspire future cultivatiors, researchers and resource developers of *S. grosvenorii*, driving wider applications in the fields of food, medicine, health products, agricultural products, and cosmetics.

## Figures and Tables

**Table:** 

**Akt**	protein kinase B
**AMPK**	5′AMP-activated protein kinase
**ATP**	adenosine triphosphate
**BALF**	bronchial alveolar lavage fluid
**BDNF**	brain-derived neurotrophic factor
**CAT**	catalase
**CDAA**	choline-deficient, methionine-deficient, L-amino acid-defined
**CDAA-HF-T (-)**	high-fat diet lacking choline, low methionine, L-amino acid restriction, and free of trans fatty acids
**CNS**	central nervous system
**COPD**	chronic obstructive pulmonary disease
**COX-2**	cyclooxygenase-2
**CSE**	cigarette smoke extract
**CUMS**	the chronic unpredictable mild stress
**DMBA**	7,12-dimethylbenz [α]-anthracene
**EBV-EA**	Epstein-Barr virus early antigen
**ECM**	extracellular matrix
**ERK**	extracellular signal-regulated kinase
**FBG**	fasting blood glucose
**GLP-1**	glucagon-like peptide-1
**GRAS**	Generally Recognized as Safe
**GSP**	glycated serum protein
**GSH-PX**	glutathione peroxidase
**GTF**	Glucosyltransferase
**HDL-C**	high-density lipoprotein cholesterol
**HO-1**	heme oxygenase-1
**HMGB1**	high mobility group box 1
**HOMA-IR**	homeostasis model assessment of insulin resistance
**IKK**	inhibitor of kappa B kinase
**IL**	interleukin
**iNOS**	inducible nitric oxide synthase
**IPF**	idiopathic pulmonary fibrosis
**IVM**	*In vitro* maturation
**IVP**	*in vitro* production
**JNK**	c-Jun N-terminal kinase
**LC-MS**	liquid chromatography-mass spectrometry
**LPS**	Lipopolysaccharide
**L-SGgly**	low-polar *Siraitia grosvenorii* glycosides
**LTB4**	leukotriene B4
**MAPK**	mitogen-activated protein kinase
**MDA**	malondialdehyde
**MF**	myocardial fibrosis
**MGEs**	mogroside-rich extracts
**MMP**	matrix metalloproteinases
**Mog**	mogroside
**mPFC**	medial prefrontal cortex
**MSG**	monosodium glutamate
**mTOR**	mammalian target of rapamycin
**M3R**	toxomatine 3 acetylcholine receptor
**NASH**	nonalcoholic steatohepatitis
**NF-κB**	nuclear factor-κB
**NMDA**	glutamate-N-methyl-D-aspartate
**Nrf2**	nuclear factor erythroid2-related factor 2
**NSG**	nanoselenium *Siraitia grosvenorii*
**OA**	Osteoarthritis
**ONOO2**	peroxynitrite
**OVA**	valbumin; p-AMPKα: phosphorylated AMP-activated protein kinase alpha
**PD**	Parkinson’s disease
**PGC-1α**	peroxisome proliferator-activated receptor gamma coactivator 1-alpha
**PGE2**	prostaglandin E2
**PPI**	prevent prepulse inhibition
**PI3K**	phosphoinositide 3-kinase
**POST**	postoperative sore throat
**UPLC-Q-TOF/MS**	ultra-performance liquid chromatography/quadrupole time-of-flight mass spectrometry
**ROS**	reactive oxygen species
***S. Grosvenorii***	*Siraitia grosvenorii*
**SGEs**	*Siraitia grosvenorii* extracts
**SG-ex**	*Siraitia grosvenorii* crude extract
**SGP**	*S. grosvenorii* polysaccharide
**SGREs**	*Siraitia grosvenorii* residue extracts
**SIRT1**	silencing message regulator 1
***S. mutans***	*Streptococcus* mutans
**SOD**	superoxide dismutase
**T2DM**	type 2 diabetes mellitus
**TCA**	cycle: the citric acid cycle
**TC**	total cholesterol
**TFAM**	transcription factor A
**TG**	triglycerides
**TLR4**	Toll-like receptor 4
**TNF**	tumor necrosis factor
**TrkB**	tyrosine kinase receptor B
**5-LOX**	5-lipoxygenase
**11-O-Mog**	11-oxo-mogroside
